# Nano drug delivery systems for advanced immune checkpoint blockade therapy

**DOI:** 10.7150/thno.112475

**Published:** 2025-04-13

**Authors:** Chenqi Guo, Ling Lin, Yihan Wang, Jing Jing, Qiyong Gong, Kui Luo

**Affiliations:** 1 Department of Radiology, Huaxi MR Research Center (HMRRC), Institution of Radiology and Medical Imaging, Rehabilitation Therapy, Breast Center, Institute of Breast Health Medicine, Frontiers Science Center for Disease-Related Molecular Network, State Key Laboratory of Biotherapy, West China Hospital, Sichuan University, Chengdu 610041, China.; 2 West China School of Medicine, Chengdu 610041, China.; 3 Functional and molecular imaging Key Laboratory of Sichuan Province, Key Laboratory of Transplant Engineering and Immunology, NHC, and Research Unit of Psychoradiology, Chinese Academy of Medical Sciences, Chengdu 610041, China.; 4 Xiamen Key Lab of Psychoradiology and Neuromodulation, Department of Radiology, West China Xiamen Hospital of Sichuan University, Xiamen 361021, China.

## Abstract

Immune checkpoint inhibitors (ICIs) have been widely utilized in the first-line therapy of various types of cancer. However, immune-related adverse events (irAEs) and resistance to ICIs remain intractable challenges for immune checkpoint blockade (ICB) therapy during clinic treatment. Nano drug delivery systems (NDDSs) have shown promising potential to improve anticancer efficacy and reduce side effects of small molecular drugs. The combination of nanotechnology and ICB provides new opportunities to overcome the challenges of immunotherapy. Nanoplatforms have been employed for direct delivery of ICIs, and they are preferred vehicles for combination therapy of ICIs and other therapeutic agents. In this review, the strategies of using NDDSs for advancing ICB therapy in recent years are surveyed, emphasizing the employment of NDDSs for combination treatment by ICIs and other agents to manipulate antitumor immunity. Analysis of current strategies for applying NDDSs for ICB leads to future research directions and development trends.

## 1. Introduction

Immunotherapy is the most attractive cancer treatment method. Especially, the emergence of immune checkpoint blockade (ICB) therapy has brought unprecedented benefits to cancer patients. Immunosuppressive signals in tumors can be manipulated through immune checkpoint pathways, thereby impairing antitumor immune responses and leading to immune escape. Cytotoxic T lymphocyte-associated protein 4 (CTLA-4) is the first discovered immune checkpoint (ICP), while programmed cell death protein 1 (PD-1) and programmed death-ligand 1 (PD-L1) are the most studied ICPs 1-3. Other ICPs, such as lymphocyte activation gene-3 (LAG-3), T cell immunoglobulin and mucin-domain containing-3 (TIM-3), T cell immunoglobin and ITIM domain (TIGIT), V-domain Ig suppressor of T cell activation (VISTA), have been revealed in recent years 4-6. So far, immune checkpoint inhibitors (ICIs) approved by FDA are monoclonal antibodies (mAbs), including anti-PD-1 mAbs (aPD-1) (nivolumab, pembrolizumab, cemiplimab, dostarlimab and retifanlimab), anti-PD-L1 mAbs (aPD-L1) (durvalumab, atezolizumab and avelumab), anti-CTLA-4 mAbs (aCTLA-4) (ipilimumab and tremelimumab), and anti-LAG-3 mAb (relatlimab), and these ICIs block the interaction between ICPs and their ligands 7. In addition, nucleic acids (such as small interfering RNA (siRNA), short hairpin RNA (shRNA), clustered regularly interspaced short palindromic repeats (CRISPR)/CRISPR-associated proteins (CRISPR/Cas), plasmid DNA, and aptamers), peptides (such as ^D^PPA-1, TPP-1, OPBP-1, and AUNP-12), small molecular drugs (such as CA-170, BMS-202, JQ1, and metformin), and DNAzyme have been used as ICIs in clinical and preclinical studies 8-10. These drugs acting as ICIs could be through the mechanism of inhibiting the expression, promoting the degradation of ICPs, or blocking their interaction with their ligands 11.

ICB has been employed to treat some types of cancer in the clinic. However, immune-related adverse events (irAEs) and resistance to ICB are two major challenges of ICIs for their clinical application 7, 12, 13. According to variations in the kinds and stages of cancer and the targets of ICIs, distinct irAEs, including more than 70 diverse pathologies, are found in all organs of the entire body, such as leukoderma, colitis, hypophysitis, pneumonia, arthritis, and myocarditis 14, 15. These irAEs can be graded from 1 to 5 based on the severity according to the Common Terminology Criteria for Adverse Events (CTCAE) 16. Low-grade irAEs occur in more than 90% of patients, while more severe irAEs, some of which may be potentially fatal, have been observed in 20%-60% of patients 17. Overactivation of the immune system and indiscriminate attack of T cells on both tumor and normal tissues are two predominant causes of irAEs 18-20. Stronger side effects are often ascribed to more effective activation of the immune system. Thus, irAEs are correlated with positive responses to ICIs 17. The scope of the immune activity can be broadened due to the widespread of ICIs in peripheral tissues outside of tumors. Therefore, blocking drug diffusion into peripheral tissues and elevating the level of ICI accumulation within tumors are crucial to relieving irAEs for ICB therapy. Meanwhile, ICI resistance, including primary resistance and acquired resistance, is another challenge for ICB. The degree of ICI resistance in diverse types of cancer is distinctly different. The response rate to single-agent aPD-1 ranges from about 40% to 70% in some types of cancer (such as melanoma, Merkel cells, Hodgkin's lymphoma), compared to only 10%-25% in other indications. Among those patients who initially respond to ICIs, a large proportion of them experience disease progression, which is considered as acquired resistance. The occurrence rate of acquired resistance varies in different types of cancer, ranging from approximately 10%-70% 21. The precise mechanisms underlying ICI resistance remain to be fully elucidated. However, it is known that complex interactions among tumor cells, the tumor microenvironment (TME), and host factors contribute to ICI resistance. These include the loss or reduction of tumor immunogenicity, dysfunction of antigen presenting cells (APCs), blockade of the antigen presentation process, metabolic adaptability of tumor cells, and excessive infiltration of immunosuppressive cells 17. Harnessing these interactions could improve the response rate and therapeutic efficacy of ICIs.

Nanotechnology has been proven to improve the physiochemical properties of therapeutic drugs. Nano drug delivery systems (NDDSs) based on nanomaterials, including nanoparticles, liposomes, micelles, and vesicles, have been utilized to incorporate therapeutic drugs to formulate nanomedicines for treating various diseases in the past few decades. Compared with therapeutic drugs without NDDSs, nanomedicines have remarkably bolstered the efficacy and compliance of antitumor agents by increasing their physic-chemical stability, improving their pharmacokinetic properties, enhancing their target tissue accumulation, and regulating their release behaviors 22-24. An array of nanomedicines have been approved for clinical application 25. In addition, many of them are in preclinical and clinical trials, demonstrating promising application prospects of nanomedicines. In the field of ICB therapy, rationally designed drug delivery systems are crucial to maximize the therapeutic efficacy of multifarious ICIs since NDDSs can directly optimize the physiochemical properties of ICIs, and they can be used for combination treatment to overcome ICI resistance 26, 27.

**Figure 1:**
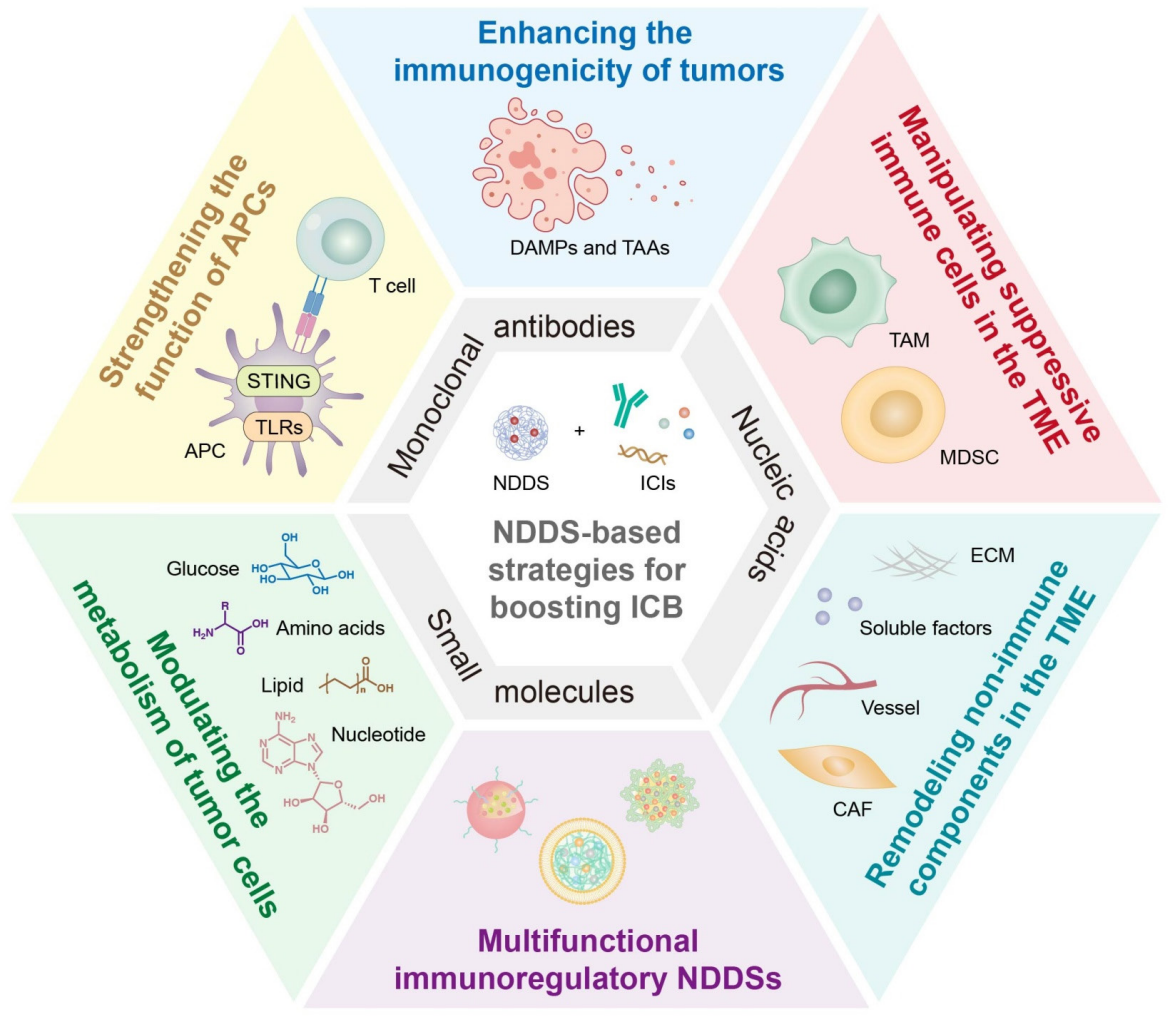
**NDDS-based strategies for boosting ICB therapeutic effects.** ICIs include monoclonal antibodies, nucleic acids, and small molecules. The combination therapy strategies include enhancing the immunogenicity of tumors, strengthening the function of APCs, manipulating suppressive immune cells in the TME, modulating the metabolism of tumor cells, remodeling non-immune components in the TME, and employing multifunctional immunoregulatory NDDSs. NDDS, nano drug delivery system; ICB, immune checkpoint blockade; ICIs, immune checkpoint inhibitors; APCs, antigen presenting cells; TME, tumor microenvironment.

In this review, we survey recent strategies for enhancing the therapeutic effect of ICIs through NDDSs **(Figure 1)**. NDDSs are employed to deliver ICIs to specifically reach target cells or improve their accumulation at the tumor site; alternatively, NDDSs are harnessed to accommodate multiple therapeutic drugs for combination therapy. We classify these nanomedicines based on their intrinsic therapeutic mechanisms. Design strategies for these nanomedicines to improve ICI efficacy and mitigate irAEs and resistance are elaborated. Moreover, future trends and challenges in developing nanotechnology-enabled ICB therapy are discussed.

## 2. NDDSs for different types of ICIs

**Figure 2:**
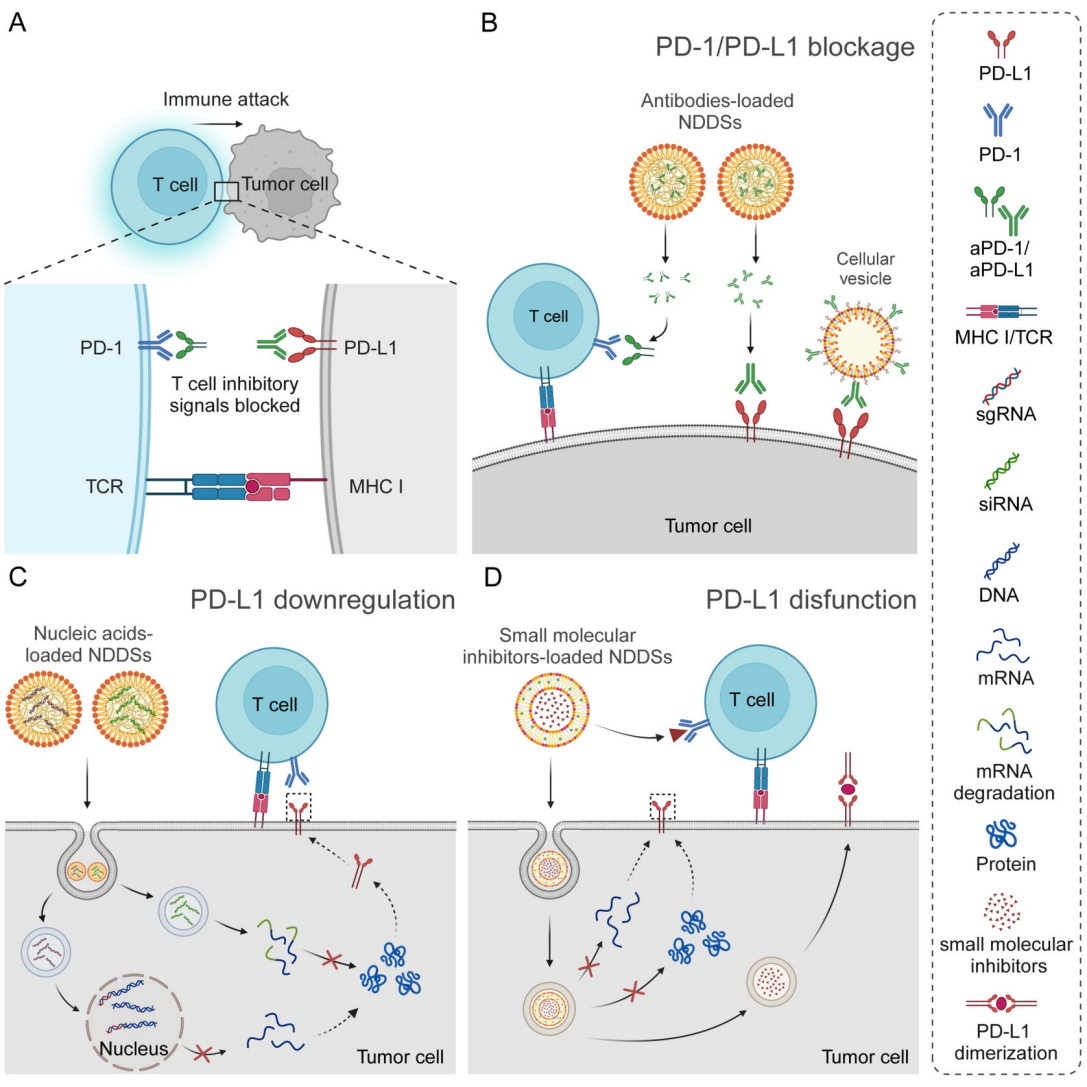
**Nano drug delivery systems (NDDSs)-based strategies for different types of ICIs.** (A) Schematic illustration for tumor cells attacked by T cells after blockage of the PD-1/PD-L1 axis. (B) Blocking the PD-1/PD-L1 axis by NDDS-delivered antibodies. (C) Downregulating PD-L1 by NDDS-delivered nucleic acids. (D) Blocking the PD-1/PD-L1 axis by NDDS-delivered small molecular inhibitors.

NDDSs have been developed for targeted delivery of various ICIs, most of which are devoted to PD-1/PD-L1 inhibitors. The PD-1/PD-L1 axis is involved in inhibiting the function of cytotoxic T cells. PD-1 is one of the co-inhibitory receptors of the CD28 superfamily, and it can be induced by an inflammatory response in T cells, especially active cytotoxic T lymphocytes (CTLs). PD-L1, an immune checkpoint protein expressed on the many normal tissue cell and tumor cell surfaces, binds to PD-1 on active T cells, thereby preventing cell recognition by CTLs, diminishing immune responses, and inhibiting CTL activities, including cytokine secretion, lymphocyte proliferation, and activation. The interaction between PD1 and PD-L1 leads to the immune evasion of tumor cells to T cells 28, 29. ICIs could restore T-cell activities by blocking the interaction between PD-1 and PD-L1, thereby enhancing their cytotoxic effects on tumor cells 30, 31. Apart from blocking interactions between immune checkpoints, the mechanisms of these ICIs have been revealed, primarily including inhibiting transcription and translation of checkpoint proteins, promoting their degradation, and inducing functional impairment **(Figure 2)**. Different types of ICIs, including antibodies, nucleic acids, and small molecule inhibitors, can be specifically delivered to tumor tissues *via* NDDS to enhance their antitumor efficacy. NDDSs have also been designed for dual ICI delivery, for example, simultaneous delivery of PD-1/PD-L1 and CTLA-4 inhibitors. The examples will be discussed in the Section on antibodies-related NDDSs.

### 2.1. Antibodies-related NDDSs

#### 2.1.1. aPD-1 and aPD-L1

Monoclonal antibodies are the earliest developed ICIs, and they have been widely used in clinical practice. They function by disrupting the interaction between the antibody and its ligand. Monoclonal antibodies as large-molecule drugs exhibit inherent limitations, such as poor permeability and suboptimal tumor targeting, resulting in "on-target but off-tumor" effects with systemic administration of aPD-L1 antibodies. This severely hampers therapeutic efficiency and causes irAEs. In order to address these challenges, NDDSs have been developed to improve their *in vivo* distribution, enhance tumor targeting ability, and increase permeability behavior 12, 17. Antibodies could be simply encapsulated in nanomaterials, such as liposomes, polymeric micelles, gold nanoparticles, and dendrimers, to reduce off-target distribution 32-35. NDDSs have been explored to encapsulate aPD-1 for targeting tumor cells and aPD-L1 for targeting naive T cells 36. Furthermore, specific tissue-targeting has been explored by modification of functional binding groups on NDDSs. For example, a glycosylated PEG-based NDDS (glucs-aPD-L1) was designed for targeted drug release in brain tumors by utilizing glucose transporter 1 (GLUT1), a highly expressed transporter in brain capillaries. The NDDS was constructed with multiple detachable PEG chains that are cleavable in the reductive TME, enabling selective drug release within the brain tissue 37. Alternative mechanisms have been employed to facilitate drug traversal across the blood-brain barrier in other studies.

#### 2.1.2. Dual ICIs

NDDSs have also been reported for dual-ICI delivery. In addition to the encapsulation of previously discussed PD-1/PD-L1 inhibitors into an NDDS, CTL-4 could be simultaneously encapsulated into the same NDDS. The primary mechanism of CTLA-4 is to block the interaction between CTL-4 on T cells and B7 molecules (CD80/CD86) on antigen-presenting cells. The binding of CTLA-4 and B7 activates inhibitory signals to inhibit excessive T cell activation and proliferation and avoid overactivation of the immune system. CTLA-4 inhibitors enhance T cell activation by disrupting the binding of CTLA-4 to its ligands, leading to enhanced antitumor immune responses. The concurrent use of PD-1/PD-L1 and CTLA-4 inhibitors may effectively boost the immune response by simultaneously increasing the T cell number and restoring their functionality, and the strategy has been applied in clinics for certain indications, and it is currently undergoing clinical studies for other indications. The use of NDDSs for dual-inhibitor delivery aims to improve targeting specificity and reduce non-targeted distribution 38, 39. They are also used for the delivery of dual inhibitors to specific tissues, such as the brain. For example, it was reported that a nanoscale immunoconjugate (NIC), P/a-CTLA-4 + P/a-PD-1, for trans-blood-brain barrier (BBB) delivery of CTLA-4 and PD-1 antibodies. Compared to the single checkpoint inhibitor-loaded NIC or free aCTLA-4 and aPD-1, P/a-CTLA-4 + P/a-PD-1 treatment resulted in a remarkable increase in the abundance of CD8^+^ T cells, NK cells, and macrophages and a significant reduction in the number of regulatory T cells (Tregs) in mice GL261 glioblastomas. The improvement in intratumoral antitumor immune responses prolonged the survival of glioblastoma (GBM)-bearing mice 40. Although a great antitumor efficacy was achieved in this study, the dual-ICIs delivery strategy has not been extensively explored, and there are very few reports on this strategy.

### 2.2. Nucleic acids-related NDDSs

#### 2.2.1. Strategies at post-transcriptional level

Nucleic acid ICIs, including siRNA, shRNA, CRISPR/Cas, plasmid DNA, and aptamers, have garnered extensive research attention. The inherent instability and susceptibility to degradation of nucleic acids offer great opportunities for the use of NDDSs. siRNA is the most investigated type of nucleic acid ICI. It suppresses the PD-1/PD-L1 axis by down-regulating PD-L1 or PD-1 expression at the post-transcriptional level. Various NDDS platforms have been developed for siRNA delivery, such as polymers, lipids, and biomolecules 27, 41, 42. siRNA for PD-1 (siPD-1) was reported to be encapsulated in noncationic soft polyphenol nanocapsules, amphiphilic triblock polymers, and dendrimer-entrapped gold nanoparticles to increase its tumor tissue penetration 43, 44 and avoid its degradation through facilitating endosomal escape of internalized siPD-1 45. A dual reactive oxygen species (ROS)/pH-sensitive siPD-L1 delivery system was attached to erythrocytes to construct a FEGCG/Zn/siPD-L1/erythrocyte system, which could enhance the stability of siPD-L1 *via* erythrocytes and promote tumor targeting accumulation *via* stimuli response 46. siPD-1-loaded liposomes were employed to deliver siPD-1 to T cells 47. Apart from direct encapsulation of siRNA, studies have explored indirect methods to generate nucleic acids. For instance, engineered bacteria that can produce RNA were developed to serve as *in vivo* "cell factories". This strategy can sustainably produce siRNA of PD-L1 within tumor cells, simplifying its manufacturing process and eliminating stringent shipping requirements 48. Moreover, a limited number of studies have investigated the structure-activity relationship. One study evaluating the delivery efficacy of several inorganic NPs for siRNA delivery demonstrated that layered double hydroxide and lipid-coated calcium phosphate had an equivalent delivery efficiency 49. Systematic investigations and meta-analyses involved in the structure-activity relationships across diverse types of NDDS for nucleic acid drug delivery remain scarce. This reveals a significant gap between the current laboratory research of nucleic acid-related NDDSs and their clinical application. In summary, various NDDSs have been extensively applied in siRNA delivery research. These NDDSs pronouncedly enhance the stability of siRNA through direct encapsulation or bypass of the endocytosis pathway to prevent degradation, resulting in reducing systemic clearance, improving tissue permeability, and enhancing tumor targeting.

#### 2.2.2. Strategies for gene editing

CRISPR/Cas is a highly efficient gene-editing tool that can be utilized to knock out PD-1/PD-L1 genes. In order to enhance the stability and delivery efficiency of nucleic acids, NDDSs have been introduced to protect them from degradation. The rational design of NDDSs for CRISPR/Cas delivery should consider multiple factors, including the choice of nanomaterials to minimize non-specific *in vivo* distribution and enhance the targeted release and an appropriate gene editing system to improve gene editing efficiency while reducing off-target effects 50. For example, a silk fiber-derived hydrogel was constructed with genetically engineered adenoviruses, which were employed to deliver CRISPR/Cas9 for PD-L1 gene editing in Hepa 1-6 liver cancer cells. The NDDS was found to promote local retention of CRISPR/Cas9 and obtain effective gene transduction over 9 days 51. Stimuli-responsive NDDSs were designed to mitigate the side effects of the CRISPR/Cas 9 system by responding to specific tumor environment characteristics 52, 53. A dual-locking NP (DLNP) was used to load CRISPR/Cas13a inside the core, and the outer shell was a dual-responsive polymer layer. The shell could promote the stability of DLNP in the circulation system to improve its biosafety, and achieve responsive release of CRISPR/Cas13a from its core in the tumor tissue 54. A remarkable efficiency in knocking out the PD-L1 gene has been achieved by optimizing of gene editing systems and nanomaterials. Gene editing systems, including CRISPR/Cas9, and CRISPR/Cas13a, have been studied for gene editing in tumor cells or T cells. Among nanomaterials, polymer nanoparticles, lipid-based nanoparticles, hydrogels, gold nanoparticles, and exosomes have been demonstrated to be promising for constructing effective NDDSs for transporting these gene editing systems.

#### 2.2.3. Other strategies

In addition to directly downregulating PD-1/PD-L1 expression, other strategies for regulating gene transcription, translation, and post-translational modification have also been developed. For instance, a lipid-protamine-DNA nanoparticle was loaded with a plasmid DNA encoding a trap protein targeting PD-L1 (PD-L1 trap). The translation of the PD-L1 trap gene generated a trivalent trap protein that had a significantly stronger degree of affinity for mouse PD-L1 than endogenous PD-1; thus, this trap protein could be used as an antagonist to inhibit the PD-1/PD-L1 axis 55. Aptamers have also been employed because of their similar functions to antibodies but better stability and modifiability. A hydrogel was reported to encapsulate aptamers. The hydrogel NDDS encapsulated an aptamer and a targeting sgRNA sequence. The PD-1 aptamer sequence could be cut by Cas9/sgRNA after the hydrogel lost its gel property for programmed release 56.

### 2.3. NDDSs for small molecular inhibitors

#### 2.3.1. Peptides

The remarkable anti-cancer efficacy of large-molecule ICIs in clinical practice with intrinsic limitations of large-molecule drugs has driven the development of small-molecule ICIs. There are two main types of small-molecule ICIs: peptides and chemical ICIs. Although these ICIs exhibit superior tissue penetration compared to large molecular ICIs, the non-specific distribution of small molecular ICIs may result in systemic toxicity, which could be addressed by employing NDDSs. Below are a few examples of NDDSs utilized in peptide ICI delivery. P-F4, a peptide identified by screening a phage display peptide library, could block the PD-1/PD-L1 axis. It was encapsulated with mPEG-PLA to improve the solubility of the peptide and avoid its rapid enzymatic degradation 57. It was also reported that an anti-PD-L1 peptide was encapsulated in a photo-induced crosslinked liposome and then conjugated on the terminal of DSPE-PEG_2k_ for PD-L1 tumor targeting. The peptide could intervene in the PD-L1 recycling mechanism, and the NDDS could achieve controlled release by promoting the intermolecular crosslinking of the liposomal bilayers to form a multivalent binding complex. The complex was transported into lysosomes instead of endosomes. Thus, the recycling of PD-L1 onto tumor cell surfaces was prevented 58.

#### 2.3.2. Chemical ICIs

Chemical ICIs are also widely studied for immunotherapy through various inhibitory pathways, including interfering with the transcription or translation of PD-1/PD-L1, reducing protein expression, promoting degradation, or competitively binding to the immune checkpoint to prevent the interaction between PD-1 and PD-L1 59. NDDSs have been explored to deliver chemical ICIs. For instance, a chemically synthesized mimicking antibody, molecularly imprinted polymer (MIP), was reported to act as a nano-sized PD-1 inhibitor. It was designed to construct the anti-PD-1 nanoMIP by epitope imprinting using the N-terminal epitope of PD-1 as the binding site 60. Notably, most peptides and chemically synthesized ICIs are currently in the laboratory/preclinical research stage. Their application requires extensive experimental validation, including druggability studies and safety investigations.

In summary, antibodies, nucleic acids, and small molecular inhibitors have been loaded into various NDDSs to improve the antitumor efficacy of these ICIs by improving their tumor targetability and accumulation. NDDSs have endowed ICIs with tumor-targeting biodistribution and strengthened their function specificity, which could contribute to reducing the incidence and alleviating the degree of irAEs 17. However, experimental/clinical evidence of relieving irAEs *via* NDDSs remains to be seen because animal models used in these studies have not shown clinically relevant adverse events, which may be due to species differences between mice and humans, the tolerance of certain strains of mice, or short-term treatment interventions in the currently available preclinical models 61. In addition, to elicit robust antitumor immune responses and diminish general toxicity, ICIs are usually combined with other therapeutic agents, which will be discussed in Section 3.

## 3. NDDSs for combination immunotherapy with ICIs

Monotherapy with ICIs is often insufficient to restore the tumoricidal function of T cells, and immune resistance is easily developed because the initiation of the antitumor immunity is strongly influenced by complicated interplays between cancer cells, the TME, and the immune system. Tumor intrinsic factors, such as low immunogenicity, lead to insufficient tumor antigen presentation. Thus, tumor cells are invisible to the immune system 62, 63. Besides, anergic lymphocytes, infiltrated immunosuppressive cells, cancer-associated fibroblasts (CAFs), dense extracellular matrix (ECM), and immunosuppressive signal/chemotaxis factors in tumor tissues constitute an immunosuppressive TME. The suppressive immune cells, including Tregs, MDSCs, and tumor-associated macrophages (TAMs) in the TME play crucial roles in tumor progression. Immunosuppressive signal factors, including vascular endothelial growth factor (VEGF) and transforming growth factor-β (TGF-β), can be secreted by neoplastic cells and other stromal cells in the TME to suppress antitumor immune responses 17, 64. In addition, unique metabolic characteristics of tumor cells contribute to the formation of an immunosuppressive TME. Hypoxia in solid tumors, caused by insufficient oxygen supply by the abnormal vasculatures and a high level of oxygen consumption by rapidly growing tumor cells, leads to the recruitment of inhibitory immune cells at tumor sites to secrete adenosine and enhance PD-L1 expression for suppression of immune responses. Low pH in the TME resulting from aerobic glycolysis and lactic acid generation of tumor cells could also inhibit the infiltration and activation of antitumor effector cells. T cell receptor expression and antigen-specific T cell response can be restrained by arginase-mediated arginine depletion, which eventually hampers immune cell infiltration and reduces their antitumor activity 65. Interaction between tumor cells and immunosuppressive TME constituents jointly induces immune escape of tumor cells, ultimately leading to uncontrolled tumor growth and an unsatisfactory antitumor effect of ICIs 13. Various NDDS-based strategies for combination therapy illustrated in **Figure 1** have been developed to boost the antitumor efficacy of ICIs, reshape the immunosuppressive TME during ICI treatment, mitigate immune resistance, and reduce irAEs after ICB treatment.

### 3.1. Enhancing the immunogenicity of tumors

#### 3.1.1. Multimodal therapy

**Figure 3:**
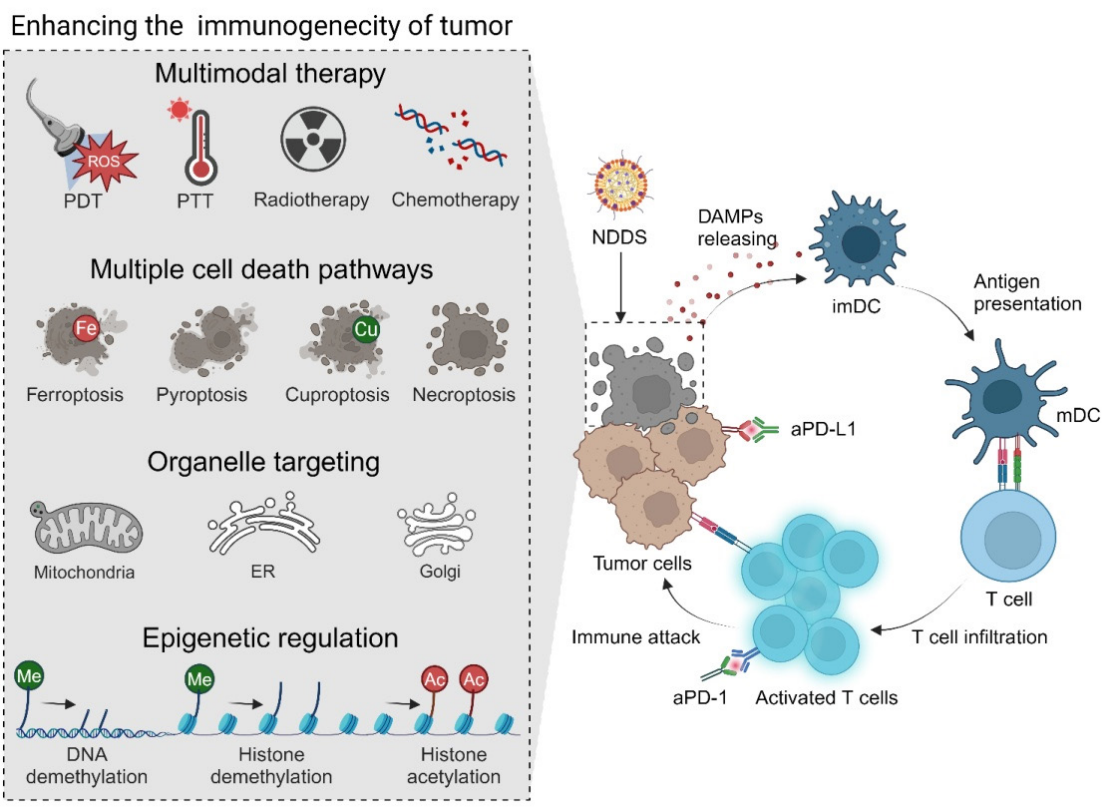
** Strategies for improving the efficacy of ICIs by enhancing the immunogenicity of tumors, including applying PDT, PTT, radiotherapy, and/or chemotherapy to induce immunogenic cell death through ferroptosis, pyroptosis, cuproptosis or necroptosis, targeting specific organelles, such as the ER and mitochondria, and performing epigenetic regulation by DNA demethylation and histone demethylation and acetylation.** ER, endoplasmic reticulum; imDC, immature dendritic cell; mDC, mature dendritic cell.

It is well known that neoplastic cells lose their immunogenicity through multiple mechanisms. A low level of immunogenicity of tumor cells allows them to avoid being recognized and captured by the immune system, which is one of the primary mechanisms of tumor immune escape and ICI resistance. Therefore, enhancing the immunogenicity of tumors appears to be an effective strategy to improve the ICB response, and it can be realized through multiple approaches **(Figure 3)**. Typically, immunogenic cell death (ICD), characterized by exposure and release of damage-associated molecular patterns (DAMPs), including calreticulin (CRT), high mobility group protein B1 (HMGB1), and adenosine triphosphate (ATP), has been regarded as a crucial event in stimulating the immune system and triggering the antitumor immunity 66, 67. Chemotherapy, radiotherapy, chemodynamic therapy (CDT), photodynamic therapy (PDT), photothermal therapy (PTT), sonodynamic therapy (SDT), and two or more combinations have been extensively used to induce ICD of tumors to improve their immunogenicity, initiate the antitumor immunity, and strengthen the therapeutic effect of ICIs 68-76. In this field, nanomaterials are usually employed to load therapeutic drugs for the above therapeutic modalities to improve their tumor-targeting ability, enhance their bioavailability, reduce their side effects, and optimize their release properties 77-80. A glutathione (GSH)-sensitive polyethylene glycol-poly-L-lysine (PEG-PLL) micelle, which was decorated with angiopep-2 peptide on the surface to allow efficient BBB penetration, was used to co-encapsulate aPD-L1 and paclitaxel (PTX) to realize effective chemo-immunotherapy of GBM 81. In another study, hollow mesoporous organosilica nanoparticles coated with the Fe metal-organic framework (MOF) that were dual-responsive to the TME were exploited to deliver doxorubicin (DOX). This nanosuspension exhibited strong tissue adhesion, killed residual tumor cells, and induced ICD *via* locally sustained release of DOX after perioperative treatment. When the DOX-loaded nanoparticles were combined with aPD-1, postoperative recurrence and brain metastasis of 4T1 breast cancer were effectively inhibited 82. In addition to commonly used cytotoxic agents, naturally-derived bioactive compounds, such as ginsenoside Rg3, quercetin 83, artesunate 84, celastrol 85, and shikonin 86, have been explored for inducing ICD and synergizing PD-1/PD-L1 blockade.

#### 3.1.2. Multiple cell death pathways

In recent years, many efforts have been devoted to inducing potent ICD through specific cell death modes, especially ferroptosis, pyroptosis, and cuproptosis. Ferroptosis is an iron-dependent cell death mode caused by GSH depletion, excessive accumulation of ROS, and lipid peroxidation in cells 87, 88. When ferroptosis induction is combined with PD-1/PD-L1 blockade, interferon-gamma (IFN-γ) released by activated CD8^+^ T cells can promote ferroptosis of tumor cells by inhibiting system x_c_^-^, establishing a positive feedback loop between ferroptosis and immune responses to enhance the synergistic effect on curbing tumor progression and metastasis 89. Therefore, the combination of ferroptosis induction and ICB may have great therapeutic potential. Although iron-based nanoparticles, such as Fe_3_O_4_ nanoparticles, zero-valent-iron nanoparticles, and ultrasmall single-crystal Fe NPs with an Fe core and an Fe_3_O_4_ shell, have been reported as nano inducers of ferroptosis in combination with ICIs, these nanoparticles exhibit an inadequate efficiency in ferroptosis induction 90-92. DOX and glycyrrhetinic acid have been reported to support iron oxide nanoparticles for enhanced ferroptosis, which in turn sensitizes the immune system to PD-1/PD-L1 blockage 93, 94. Furthermore, biocompatible iron-based metal-phenolic networks (MPNs) can serve as iron sources for triggering ferroptosis, as well as nanocarriers to deliver other drugs for promoting ferroptosis. In this context, sonodynamic, photodynamic, and photothermal therapeutic modalities, as well as glucose oxidase (GOx) and CO prodrugs, have been used to enhance iron-triggered ferroptosis of tumor cells by elevating the production of ROS, thus improving the antitumor effect of ICIs 95-98. Other metals have been explored to replace iron and induce ferroptosis. For example, a PEGylated Cu_2_WS_4_ nanozyme (CWP) was synthesized to trigger ferroptosis *via* the KEAP1/NRF2/HMOX1/GPX4 pathway and simultaneously assisted in radiation dose deposition in 4T1 cells, and CWP was applied in combination with a PD-L1 antibody 99. in another study, arsenic trioxide was exploited to induce ferroptosis. After integrating the photothermal effect of AuNPs, the release of tumor-associated antigens (TAAs) was significantly enhanced, thus increasing the efficacy of aPD-L1 100. In addition to metal-based inducers, small molecular ferroptosis inducers, such as erastin and (1S,3R)-RSL-3 (RSL-3), have been used in NDDS-based combination therapy with ICIs 101, 102. Moreover, ferroptosis resistance mechanisms have been harnessed to optimize ICD induction and sensitize ICB. A biomimetic nanoplatform (PMVL) was constructed through the self-assembly of tannic acid and vanadium oxides, encapsulation of lonidamine (LND), and coating with B16F10 cell membranes. The nanoplatform was then modified with an anti-PD-L1 peptide (PPA). In this nanosystem, LND-sensitized vanadium (V^IV^ and V^V^)-induced ferroptosis by reduced nicotinamide adenine dinucleotide phosphate (NADPH) and ATP in cells through glycolysis inhibition, and PPA was released in response to matrix metalloproteinase-2 (MMP-2) in the TME to block PD-1/PD-L1 recognition. Eventually, PMVL exhibited a pronounced antitumor efficacy in B16F10 tumor-bearing mice 103. The resistance to ferroptosis induction can be circumvented by inhibiting ferroptosis suppressor protein 1 (FSP1) 104. Remarkably, spontaneous ferroptosis of neutrophils in the TME has been reported to impair T cell activity *via* releasing oxidized lipids, revealing diverse roles of ferroptosis in the TME 105. A liposome was prepared to co-encapsulate a di-iodinated photosensitizer IR780 (Icy7) and a ferroptosis inhibitor, liprostatin-1, to trigger ICD of tumor cells through PDT but suppress ferroptosis of neutrophils, and this synergistic strategy enhanced the therapeutic effect of aPD-1 in gastric cancer 106.

Pyroptosis is a programmed cell death mode mediated by proteolytic cleavage and N-terminal domain exposure of gasdermin (GSDM). It is characterized by pore formation on the plasma membrane and release of proinflammatory substances, which can initiate strong immune responses. Thus, it can be combined with ICIs to achieve potent antitumor immunotherapeutic effects 107, 108. Recently, nanoengineered DOX (a chemotherapeutic agent) 109, indocyanine green (ICG, a photothermal photosensitizer) 110, YBS (a photodynamic photosensitizer) 111, phthalocyanine (a sonosensitizer) 112, Cinobufagin (an active ingredient of traditional Chinese medicine) 113, Fe- and Cu-incorporated hollow carbon spheres (a nanoenzyme) 114, have been reported to escalate inflammatory responses by inducing pyroptosis of tumor cells, effectively improving the efficacy of immune checkpoint inhibition. Considering an increased expression level of cyclooxygenase-2 (COX-2) in cancer cells after pyroptosis induced by platinum-based drugs, an amphiphilic polymer nanoparticle (Pt-In NP) was developed after co-loading a platinum prodrug and indomethacin, a COX-2 inhibitor. The Pt-In NP combined with aPD-L1 effectively restrained primary and distant tumor growth in pancreatic cancer 115. Moreover, direct expression of the N-terminal domain of GSDM (GSDM^NT^) through recombinant adeno-associated virus (rAAV) or mRNA lipid nanoparticles were reported to induce pyroptosis and sensitize tumors to checkpoint immunotherapy 116, 117.

Cuproptosis is a recently discovered mode of cell death in 2022. The main process of cuproptosis includes binding of excess copper to lipoylated components in the mitochondrial tricarboxylic acid (TCA) cycle, lipoylated protein aggregation, and iron-sulfur cluster protein reduction, ultimately resulting in proteotoxic stress and cell death 118. Similar to ferroptosis, cuproptosis can be recognized as ICD that promotes the release of TAAs and initiates antitumor immune responses 119, 120. Elesclomol (ES), a copper ionophore, can specifically transport copper to the mitochondria. It has been reported for mitochondrion-targeting delivery of Cu(II) to induce cuproptosis in neoplastic cells. Its combination therapy with checkpoint antibodies was applied to treat melanoma and bladder cancer 121, 122. In addition, cuproptosis is induced principally after interference with the TCA cycle, while tumor cells are insensitive to cuproptosis induction in a hypoxic TME since they preferentially rely on glycolysis rather than oxidative phosphorylation (OXPHOS). Therefore, siRNA for catalase and pyruvate dehydrogenase kinase 1 were incorporated into cuproptosis nanoinducers to sensitize cuproptosis by alleviating hypoxia or inhibiting glycolysis, respectively. As a result, effective anabatic cuproptosis was realized, and the antitumor efficacy of aPD-L1 was also enhanced 123, 124. Promising results of triggering antitumor immune responses after cuproptosis warrant future efforts into exploring cuproptosis inducers and their combination with ICIs in cancer immunotherapy. It is worth noting that randomly distributed copper ions in the body can cause side effects. It is crucial to improve the efficiency of targeted delivery of copper ions to tumor cells. In addition, the efficiency of cuproptosis induction in tumor cells can be compromised by a high concentration of GSH that chelates copper ions and the copper efflux mediated by ATPase copper transporting alpha/beta (ATP7A/B) 125, 126. NDDSs could play a role in mitigating these issues, paving the way for the application of cuproptosis for cancer immunotherapy.

#### 3.1.3. Organelle-targeting NDDSs

**Figure 4:**
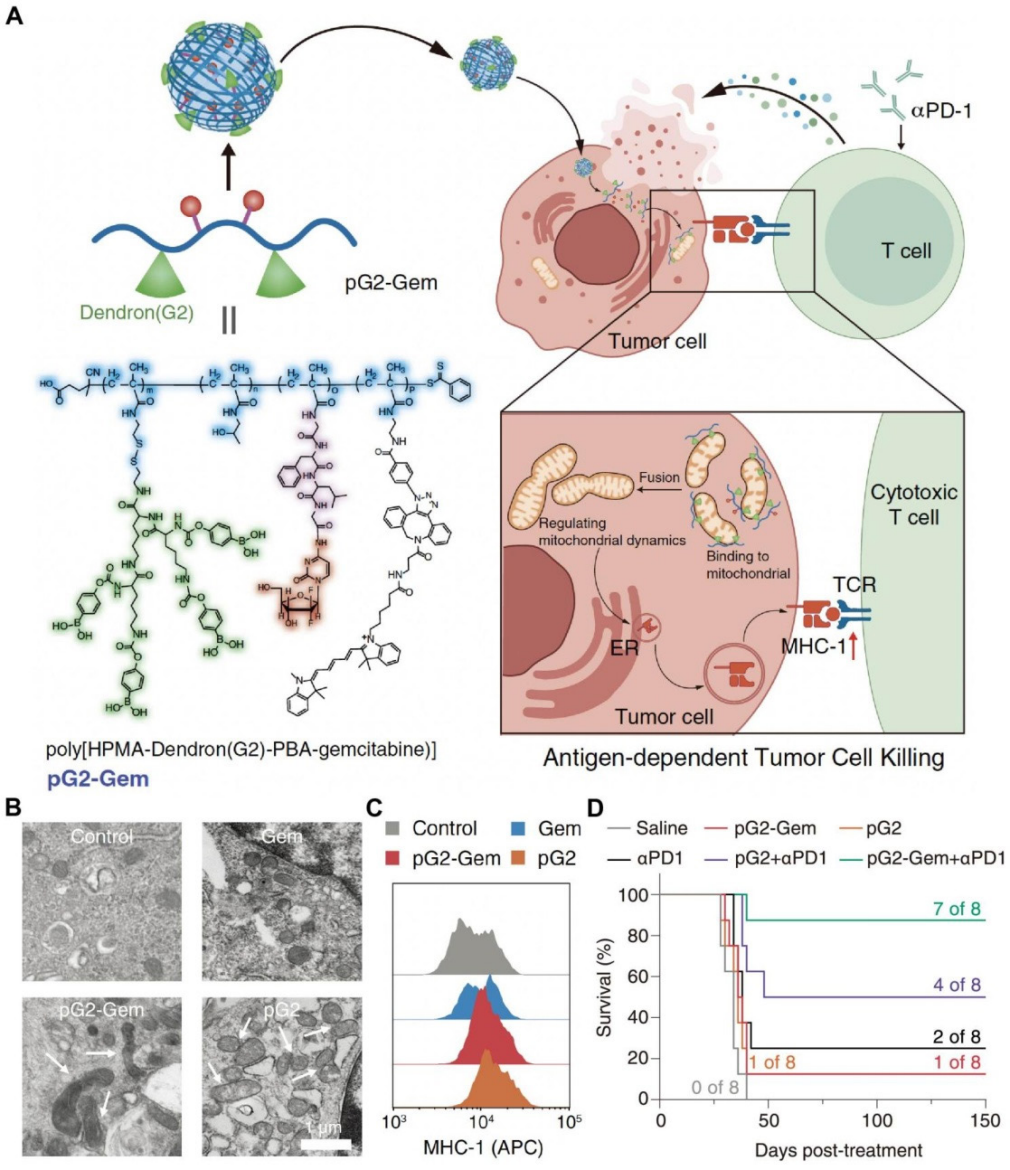
** An NDDS derived from a mitochondrion-targeting dendronized polymer for synergic therapy with aPD-1 by promoting mitochondrial fusion and enhancing the expression of major histocompatibility complex (MHC)-I.** (A) pG2-Gem released gemcitabine to exert its cytotoxic effects and the drug-free polymer, pG2, to regulate mitochondrial dynamics and promote mitochondrial fusion, thereby mediating MHC-I antigen presentation and leading to the activation of cytotoxic T cells for effective synergic therapy with aPD-1. (B) TEM images of mitochondrial fusion in 4T1 tumor cells treated with pG2 and pG2-Gem. Free Gem did not impact the mitochondrial morphology (Scale bar = 1 μm). (C) Expression levels of MHC-I in 4T1 cells after treatment with Gem, pG2, and pG2-Gem. (D) Kaplan-Meier survival curves of the 4T1 tumor model after various therapies. Reproduced with permission from ref 139. Copyright 2024, Wiley.

Compared with traditional tumor-targeting drug delivery systems that recognize and bind to surface receptors, organelle-targeting NDDSs are designed to deliver drugs to specific organelles and accurately regulate their biological functions within the organelles, which is conducive to improving efficacy and reducing toxicity 127-129. Moreover, the dysfunction of specific organelles contributes to cell death and DAMP release. It is well known that endoplasmic reticulum (ER) stress and ROS production are essential for ICD induction. ER-targeting drug delivery systems have been developed to efficiently induce ER stress. Drugs have been delivered to the ER for ICD induction and combination therapy with ICIs, including chemotherapy agents, sonosensitizers, and AIE photosensitizers 130-132. Meanwhile, the mitochondrion is an important organelle to produce intracellular ROS. Thus, mitochondrion-targeting nanodrugs have also been widely studied. To date, DOX 133, fenofibric acid 134, dichloroacetate 135, LND 136, self-assembling peptides 137, and AIE photosensitizers 138 have been conjugated with mitochondrion-targeting moieties or encapsulated into mitochondrion-targeting nanoplatforms for advancing ICB treatment. In our group, we designed and prepared a mitochondrion-targeting drug-free dendronized polymer (pG2) **(Figure 4)**. The polymer promoted mitochondrial fusion, resulting in enhanced expression of major histocompatibility complex (MHC)-I. The treatment by the dendronized polymer conjugated with gemcitabine (pG2-Gem) and aPD-1 exhibited a potent antitumor efficacy in a 4T1 mouse model 139.

#### 3.1.4. Epigenetic regulation

Epigenetic alterations play a critical role in tumorigenesis and progression, and epigenetic modifiers, such as DNA methyltransferase (DNMT) inhibitors, histone deacetylase (HDAC) inhibitors, and histone methyltransferase (HMT) inhibitors, could be promising antitumor therapeutic agents. They have been explored to directly kill tumor cells, dampen hypermethylation of tumor suppressor gene promoters, enhance expression of TAAs and MHC class I and II molecules, and alleviate T cell exhaustion. The revealed antitumor mechanisms of these drugs imply their potential to improve ICB therapeutic efficacy 140, 141. Zebularine 142, 143, Panobinostat 144, chidamide 145 and SAHA 146 have been incorporated in NDDSs for combination therapy with PD-1/PD-L1 blockade. Decitabine 147, olsalazine 148, 5-azacytidine 149, and CM-272 150 have been applied to sensitize nanoplatform-based chemotherapy or SDT for amplified ICD and effective synergistic influence with PD-1/PD-L1 antibodies.

### 3.2. Strengthening the function of APCs

**Figure 5:**
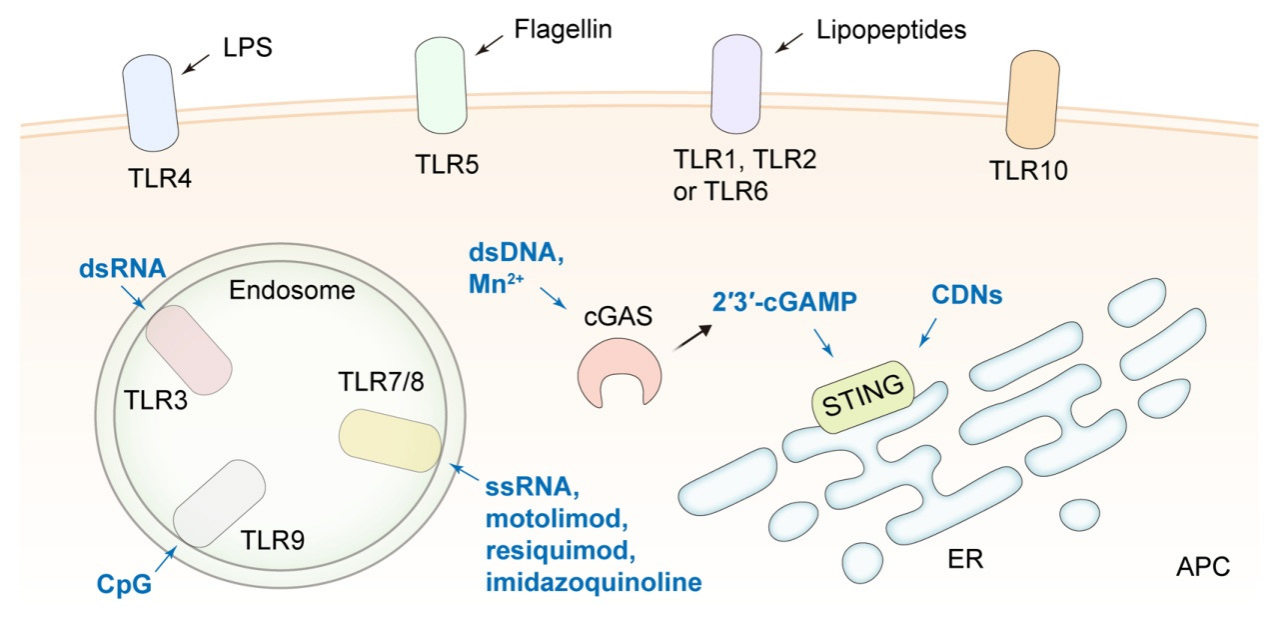
**TLR and STING agonists delivered by NDDSs for strengthening the function of APCs and improving the efficiency of ICB.** TLR, toll-like receptor; cGAS, cyclic guanosine monophosphate-adenosine monophosphate synthase; STING, stimulator of interferon genes; 2′3′-cGAMP, 2′3′-cyclic guanosine monophosphate-adenosine monophosphate; CDNs, cyclic dinucleotides; dsRNA, double-stranded RNA; dsDNA, double-stranded DNA; LPS, lipopolysaccharide; ER, endoplasmic reticulum; APC, antigen presenting cell.

APCs, predominantly DCs and macrophages, play a crucial role in the process from the release of tumor antigens to the induction of antitumor immune responses, including antigen processing and presentation and T cell recruitment and activation 151, 152. The function of APCs is significantly impacted by their surrounding environmental factors. The immaturity of DCs in an immunosuppressive TME severely curtails specific antitumor immunity 153. Therefore, various immune stimulants, such as toll-like receptor (TLR) agonists, cytokines, chemokines, and STING agonists, have been applied to improve the therapeutic effect of ICB by strengthening the function of APCs **(Figure 5)**.

CpG oligonucleotides 154, 155, imiquimod (R837) as a TLR7 agonist 156, 157, resiquimod (R848) 158-161, imidazoquinoline 162 and motolimod 163 as TLRs 7/8 agonists have been delivered by DNA tetrahedrons, polymer-based nanoparticles, liposomes, nanogels and MOFs, respectively, for APC activation in tumor tissues and combination therapy with ICIs. For example, a bispecific nano-immunoengager (NIE) that was assembled from two transformable peptide monomers was reported. One monomer consisted of a tumor-targeting LXY30 cyclic peptide, a β-sheet-forming peptide, and a hydrophobic pheophorbide a (Pa) moiety. Another monomer was composed of a "pro-ligand" form of LLP2A that targeted the activated α_4_β_1_ integrin on lymphocytes, a β-sheet-forming peptide, and R848. The two monomers could co-assemble into NPs with a diameter of about 28 nm (NIE-NPs) when they were mixed at a ratio of 1:1. After binding to the α_3_β_1_ integrin of tumor cells, NIE-NPs formed a nanofibrillar structural network on cancer cells. Under the action of esterase in the TME, LLP2A converted from proLLP2A captured CD8^+^ T cells by recognizing the activated α_4_β_1_ integrin, and R848 released from NIE stimulated the presentation of antigens, the release of antitumor response factors, and the transformation of the macrophage phenotype from M2 to M1. The NIE significantly improved the efficacy of PD-1/PD-L1 ICB therapy in two different cancer models in mice 158. A tumor-colonized attenuated *Salmonella typhimurium* VNP20009 strain was engineered to synthesize granulocyte-macrophage colony-stimulating factor (GM-CSF) and interleukin 7 (IL-7), which helped recruiting macrophages and DCs and enhancing T cell antitumor responses. The GM-CSF-IL-7-VNP20009 strain combined with a PD-1 antibody synergistically suppressed the progression and metastasis of B16F10 melanoma 164. In addition, synthetic materials may possess immunostimulating functions that assist in ICB therapy. A phosphorus dendrimer (AK128) was reported to promote the proliferation of NK cells. The nanocomplexes were comprised of AK128 and aPD-1 and camouflaged with M1-type macrophage cell membranes. The encouraging therapeutic effect after applying the nanocomplexes to treat glioma suggested the polymers could be novel immunostimulants 165.

The cyclic guanosine monophosphate-adenosine monophosphate synthase (cGAS)-stimulator of interferon genes (STING) pathway, another innate immune pathway, has received great attention for improving the therapeutic effect of ICB. After cGAS recognizes DNA fragments in the cytoplasm, 2′3′-cyclic guanosine monophosphate-adenosine monophosphate (2′3′-cGAMP) is synthesized to activate STING, promoting the production of type I interferon (IFN-I) and other proinflammatory cytokines 166-168. Activation of the STING pathway in the APCs is essential to initiate the anticancer effect of CD8^+^ T cells. Cyclic dinucleotides (CDNs) are the most widely studied STING agonists. There is a very low level of internalization of CDNs by tumor cells due to their hydrophilicity and electronegativity, and they could induce T cell apoptosis and enhance tumor cell tolerance to ICB 169. A myriad of lipid-based nanoplatforms have been developed for the efficient delivery of CDNs. For example, a CDN-loaded and phosphatidylserine (PS)-coated liposome (LNP-CDN) was reported 170. In LNP-CDN, CDN was complexed with calcium phosphate to ensure the release of CDN from endosomes to the cytosol. LNP-CDN was demonstrated to be primarily uptaken by phagocytes, i.e., macrophages, CD103^+^ DCs, and CD11b^+^ DCs, in both malignant pleural effusion (MPE) and pleural tumors in the pleural cavity. By remodeling the phenotype of myeloid cells and enhancing the cytotoxicity mediated by CD8^+^ T cells and NK cells, LNP-CDN significantly boosted the antitumor immune response to anti-PD-L1 therapy in both mouse and human MPE. Moreover, liposomal CDN decorated with a Clec9a targeting peptide for specifically activating CD103^+^ DCs was reported to amplify the efficacy of aPD-L1 171. In another study, a CD44×PD-L1/CD3 trispecific T-cell nanoengager loaded with c-di-AMP (CDA) was designed to form an immunological cytolytic synapse between triple-negative breast cancer (TNBC) cells and T cells, leading to TNBC cell lysis, therefore, the nanoengager exhibited an outstanding antitumor efficacy 172.

In addition to CDNs, double-stranded DNA (dsDNA) can be nanoengineered to activate cGAS-STING signaling during combination therapy with ICIs 173, 174. Interestingly, nanoscale vesicles containing plant-derived mitochondrial DNA (mtDNA) isolated from *Artemisia annua* were shown to activate cGAS-STING and reshape TAMs to their proinflammatory phenotype, thereby enhancing ICB therapeutic effects 175. Nanoplatforms have been developed to selectively trigger DNA double-strand breaks (DSBs) or mtDNA leakage to stimulate the cGAS-STING pathway for combination therapy with ICIs. The reported nanoplatforms include β-lapachone and tirapazamine co-loaded liposomes anchored with a TIGIT block peptide and coated with erythrocyte membranes 176, CCR2 antagonist-incorporated ultra-small-sized micelles based on gemcitabine-conjugated polymers 177, cisplatin prodrug (Pt(IV))/WEE1 inhibitor (MK1775) co-encapsulated nanoparticles 178, and peptide nanodrugs composed of poly(2-(diisopropyl amino) ethylmethacrylate) (PDPA) conjugated with an antimicrobial peptide (AMP, KLAKLAK_2_) and a PD-L1 antagonist peptide (CVRARTR) 179.

Manganese (Mn) has been reported to play a critical role in innate immune activation *via* the cGAS-STING pathway 180. It could also catalyze ROS production through a Fenton-like reaction for cancer CDT and act as a contrast agent to enhance magnetic resonance imaging (MRI) 181. Hence, nanoformulations containing Mn^2+^ have been extensively explored in recent years. In order to reduce toxicity and increase tumor accumulation of free Mn^2+^, iRGD-modified hollow mesoporous silica 182, hollow rough MnO_2_ 183, silk sericin and pentapeptide CREKA 184, zeolite imidazole framework (ZIF-8) 185 and bioactive glass 186 have been employed to construct Mn^2+^ delivery systems for synergistic therapy with ICIs. Furthermore, mutant p53 (mutp53) proteins inhibit the STING downstream pathway by binding to TANK-binding kinase 1 (TBK1), while mutp53 can be degraded by Zn^2+^ through proteasome ubiquitination, thus mitigating the inhibitive effect by mutp53. A ZIF-8@MnO_2_ nanoparticle was proposed to deliver Mn^2+^ and Zn^2+^, and the immunotherapeutic efficacy of ZIF-8@MnO_2_ combined with aPD-L1 in tumors with mutated p53 was verified 187. For synergetic STING activation, Mn^2+^ collaborated with CDNs in nanoplatforms to enhance the potency of ICIs 188, 189. In addition, an ultra-pH-sensitive and TME-targeting NDDS was designed by encapsulating a TLR4 agonist MPLA and manganese tetroxide nanoparticles into polyethylene glycol-poly(ethylpropylaminoethyl methacrylate). Since nuclear factor-κB (NF-κB) activation mediated by the TLR4 agonist amplified the magnitude of STING activation, this NDDS synergizing with aPD-1 induced tumor regression and established systemic antitumor memory 190.

Cancer vaccines, which directly activate APCs to trigger tumor-specific immune responses, have exhibited great potential in immunotherapy 191. Especially, nanovaccines can efficiently deliver antigens to APCs, improve cytosolic retention of antigens, protect antigens from degradation during the delivery process, and manipulate spatiotemporal codelivery of antigens and adjuvants compared with traditional vaccines. Therefore, they can be employed to improve the efficacy of ICB therapy 192, 193. Nanovaccines often consist of antigens, adjuvants, and carriers. Ovalbumin (OVA) and its derived antigenic peptides are the most commonly used model antigens in nanovaccines for combination therapy with ICIs **(Table 1)** 194-202. In addition, antigenic peptides identified from cancer cell lines, as well as tumor cell membranes or tumor cell lysates, have been utilized as antigens to construct nanovaccines for preclinical and clinical studies 203-206. Importantly, due to the heterogeneity and intricacy of cancer, antigens with a high degree of universality and immunogenicity remain to be discovered. Adjuvants in vaccines are essential for bolstering the immune response triggered by antigen stimulation. Many adjuvants employed in antitumor nanovaccines, such as CpG, R848, dsDNA, CDNs, and Mn^2+^, have been discussed in the previous section. Currently, very few adjuvants are approved for clinical application, and novel translatable adjuvants are actively sought for antitumor vaccines. Various carriers used in nanomedicines are also exploited for nanovaccines, such as proteins 207, 208, lipids 194, 199, 209, polymers 195, 200, 203, 210, 211, inorganic materials 204, 212, 213, cell membranes 214-217, and extracellular vesicles (EVs) 197, 218-220. It is feasible to prepare carrier-free nanovaccines by harnessing the characteristics of antigens and adjuvants through a self-assembly process 221. Particularly, a few delivery vehicles have been reported to have similar properties as adjuvants, and they can assemble with antigens into nanovaccines that initiate potent immune activation and boost ICB efficacy in cancer models 198, 222-225. It is worth noting that the efficiency of antigen cross-presentation is crucial for the efficacy of nanovaccines. The efficiency, generally characterized by examining the SIINFEKL-H-2K^b^ expression level on APCs when OVA is used as a model antigen or the T cell activation level for non-OVA antigens, is highly dependent on the lysosomal escape of the antigen or circumvention of the lysosomal endocytosis pathway. In addition to detecting immune cell activation in lymph nodes, the infiltration level of immune cells in tumor tissues is extremely important for assessing the effect of antitumor immunity.

Nanomedicines containing ICD inducers and immune adjuvants can be promising candidates for enhancing the therapeutic efficacy of ICIs since they share a mechanism similar to that of nanovaccines. The drugs discussed in Section 3.1 can be used as ICD inducers in the nanomedicines to induce ICD, promote TAA release, and enhance tumor immunogenicity. The immune stimulants mentioned above can be used as adjuvants in nanomedicines. Compared with specific antigens used in nanovaccines, the nanomedicines can trigger ICDs to generate a broader spectrum of tumor antigens, which may be beneficial to stimulate stronger immune responses 240. For example, a nanosystem (mB4S) was constructed to deliver epirubicin (EPI) as an ICD inducer and diABZI as a STING agonist, which potentiated the potency of aPD-L1 in both 4T1 and CT26 tumor-bearing mice 241. A CpG-loaded liposome was designed to incorporate 2,3-bis(((5Z,8Z,11Z,14Z)-icosa-5,8,11,14-tetraenoyl)oxy)propyl (2-(trimethylammonio)ethyl) phosphate (DAPC), a tailored phospholipid that acted as a ferroptosis inducer, and this liposome was administrated in combination with aPD-L1 242. PARE NPs were reported to be assembled from a prodrug synthesized by conjugating R848 with a photosensitizer pyropheophorbide-a, and the nanoparticles markedly inhibited the progression of distant tumors in a subcutaneous HNSCC tumor mouse model when they were combined with aPD-1 243.

### 3.3. Manipulating suppressive immune cells in the TME

**Figure 6:**
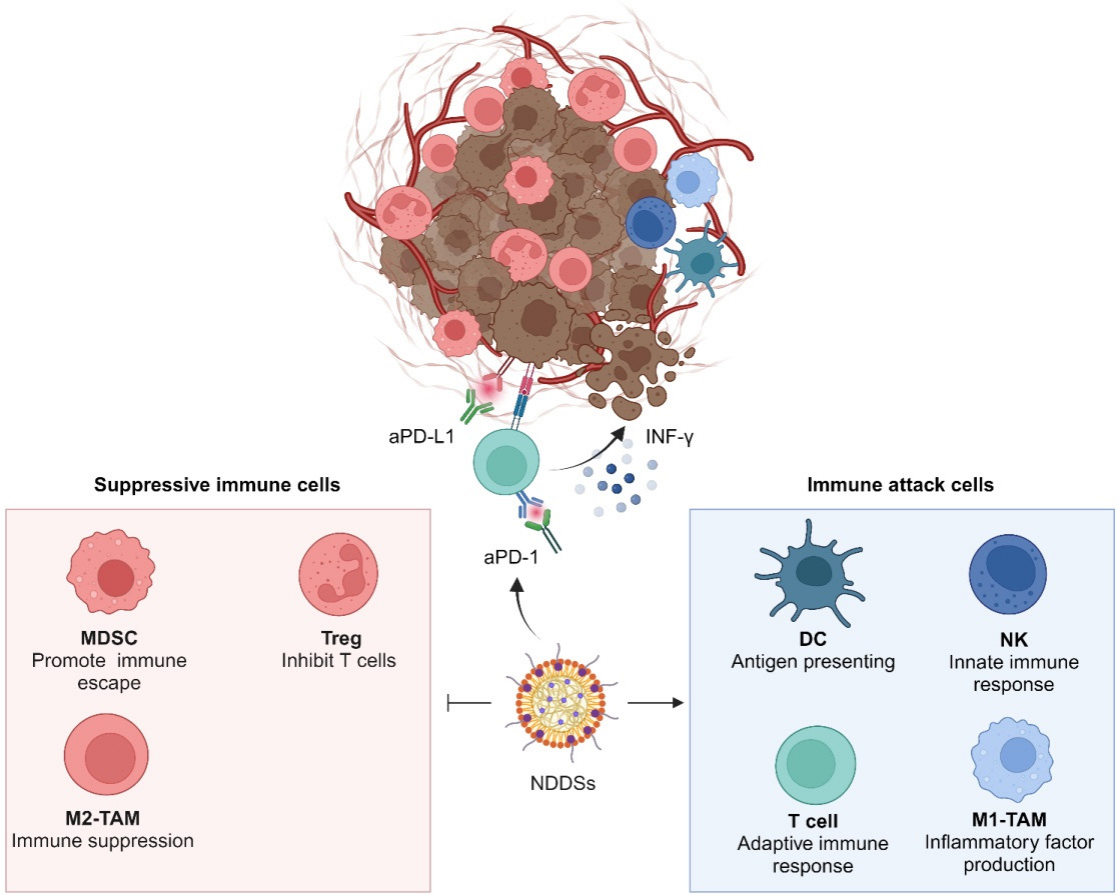
Suppressive immune cells in tumor tissues, including Tregs, M1-TAMs, and MDSCs, abate synergetic immune attacks of DCs, T cells, NK cells, and M1-TAMs.

Suppressive immune cells in tumor tissues, including Tregs, TAMs, and MDSCs, are key players in maintaining an inhibitory TME. These cells impair the function of effector T cells (Teffs) and severely diminish immune responses to ICB **(Figure 6)**. Currently, NDDSs for sensitizing ICIs by regulating immunosuppressive cells predominantly target TAMs and MDSCs.

TAMs, the most abundant immune cells in TME, exhibit an anti-inflammatory M2 phenotype, which promotes tumor progression and maintains an immunosuppressive TME by secreting anti-inflammatory factors, such as TGF-β, and IL-10 244. Therefore, efforts have been made to strengthen the efficacy of ICB by repolarizing TAMs to their M1 phenotype through regulators in NDDSs. A supramolecular peptide amphiphile drug-delivery system (SPADS) was reported. In SPADS, mannose was modified to target M2-TAMs, and toyocamycin and α-tocopherol were employed to reprogram M2-TAMs by inhibiting ER stress and oxidative stress 245. Similarly, a KIRA6 (an ER stress inhibitor) and α-tocopherol (an oxidative stress inhibitor) co-loaded nanoemulsion was developed 246. Both of the nano-formulations improved the efficacy of aPD-1 *via* repolarizing TAMs. Mannose-containing precursor glycopeptides that multivalently bound to mannose receptors on M2-TAMs and gold nanoparticles (Au NPs) coated with a polyaniline-based glyco structure were revealed to promote TAM M1-polarization and boost aPD-1 treatment effects, respectively 247, 248. In addition, TLR agonists, proinflammatory cytokines, and kinase inhibitors have been reported to reprogram the antitumor activity of macrophages for combination therapy with ICIs 249-252. M2-TAMs as APCs exhibit rather weak antigen cross-presentation due to an elevation in the lysosomal cysteine protease activity. To strengthen antigen cross-presentation of M2-TAMs, MSNs loaded with E64, a cysteine protease inhibitor, were developed. The E64-loaded MSNs were coated with surgical tumor-derived cancer cell membranes decorated with galactose ligands to target M2-TAMs by binding their CD302 receptors. Treatment with ME@C significantly improved antigen cross-presentation of M2-TAMs by inhibiting the activity of cysteine proteases and delivering abundant TAAs from the cancer cell membranes, effectively suppressing the progression and recurrence of tumors 253.

**Figure 7:**
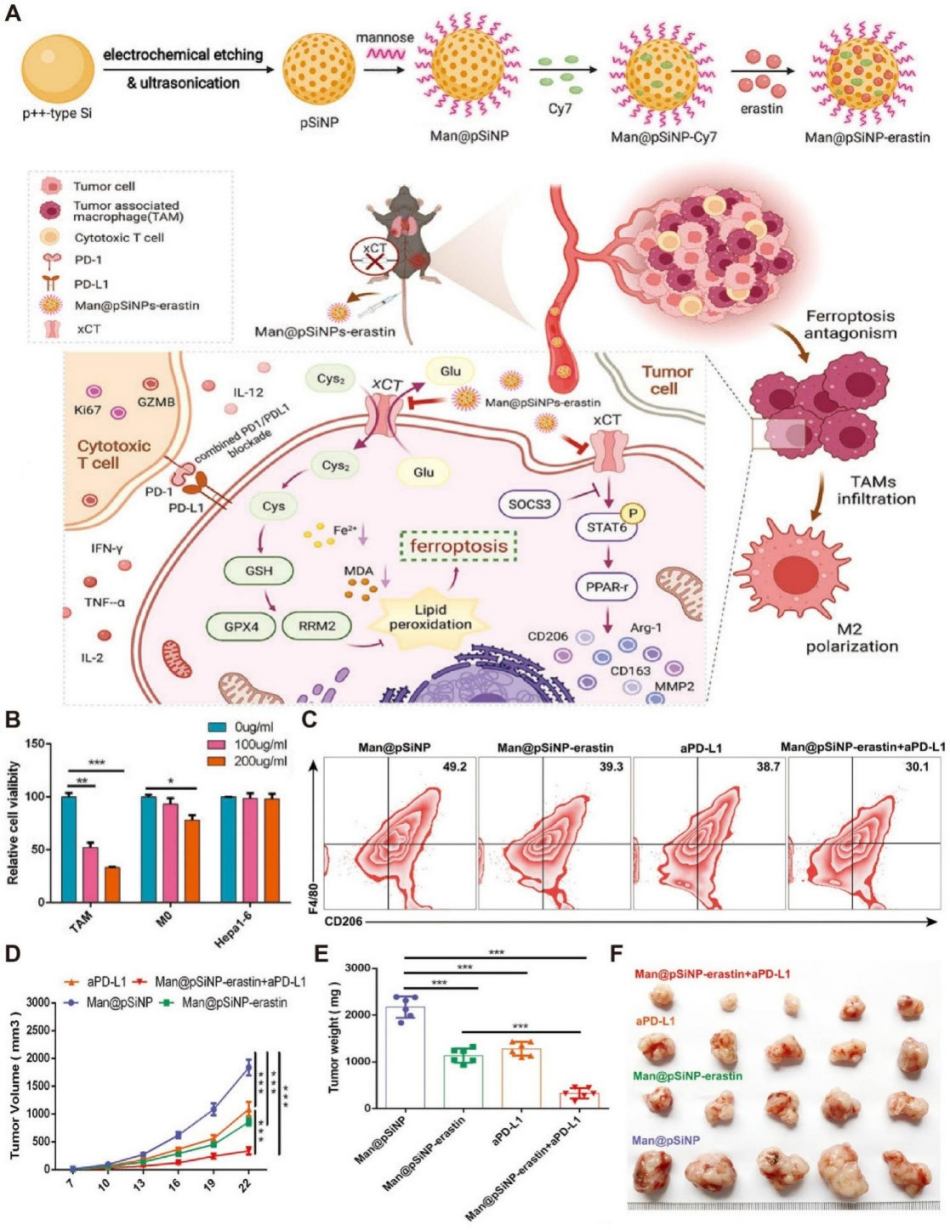
** Manipulating suppressive immune cells in the TME by NDDS-based nanomedicines to boost immunotherapeutic effects of ICIs.** (A) Schematic illustration of the preparation of Man@pSiNP-erastin and the mechanism of boosting immunotherapeutic effects using Man@pSiNP-erastin+aPD-L1. Man@pSiNP-erastin enhanced the antitumor efficacy of aPD-L1 by inducing TAM ferroptosis and reducing M2-like transformation. (B) Man@pSiNPs-erastin exhibited higher cytotoxicity on TAMs than M0 cells and Hepa1-6 cells in a dose-dependent manner (n = 3). (C) FCM analysis revealed a significantly decreased proportion of infiltrating M2-like cells in tumor tissues after treatment with Man@pSiNP-erastin+aPD-L1. (D) Growth curves, (E) tumor weights, and (F) photos of Hepa1-6 tumors after different treatments (n = 6). Reproduced with permission from ref 254. Copyright 2023, Wiley.

Although ferroptosis in tumor cells has been widely studied, ferroptosis of TAMs is rarely reported. It has been revealed that overexpressed xCT in macrophages encoded by SLC7A11 regulated M2 polarization of TAMs mediated by IL-4 and participated in activating the SOCS3-STAT6-PPAR-γ pathway. Ferroptosis induction by xCT knockout in macrophages was associated with GPX4/RRM2 signaling. Encouraged by these findings, mannose-functionalized porous silicon nanoparticles were loaded with erastin, a ferroptosis inducer, to form Man@pSiNPs-erastin. The resulting nanodrug achieved specific ferroptosis activation in M2-TAMs **(Figure 7)**, which dramatically impeded tumor progression in hepatocellular carcinoma (HCC) when it was combined with aPD-L1 254.

In addition to TAMs, MDSCs are another major type of inhibitory immune cells in the TME, and they collaborate with TAMs to maintain an immunosuppressive TME 255. Au NPs at a size of 30 nm were reported to block NLRP3-NEK7 interaction by scavenging ROS, suppressing the activity of NLRP3 inflammasomes, and blocking the release of IL-1β in myeloid cells. The authors coupled Au NPs with H6, an MDSC-targeting peptide, to improve the efficacy of aPD-1-mediated immunotherapy. The combination of Au-H6-NPs and aPD-1 significantly diminished the release of IL-1β, reduced the population of MDSCs in the TME, and promoted tumor infiltration of CD8^+^ T cells in both aPD-1 sensitive and insensitive cancer models 256. In addition, MDSCs have been demonstrated to be populated in residual tumor tissues of HCC after insufficient radiofrequency ablation (iRFA), and compensatory upregulation of PD-L1 on residual MDSCs could be achieved during combination therapy with iRFA and MDSC inhibition. A size-tunable acid-sensitive perfluorohexane-cored liposome (LPIP), which released IPI549 and aPD-L1 in response to the acidity in the TME and mild heat under iRFA, was designed. Due to a high expression level of the gamma isoform of phosphoinositide 3-kinase (PI3Kγ) in MDSCs, IPI549, a PI3Kγ inhibitor, selectively mitigated immune suppression of MDSCs. Finally, the combination of IPI549 and aPD-L1 effectively mitigated post-iRFA relapse and hindered the progression of HCC 257.

Since both MDSCs and TAMs play important roles in the formation and maintenance of an immunosuppressive TME, and they directly interact with each other in a complex way to jointly promote the immune escape of cancer cells, the simultaneous intervention of both types of immune cells has been proposed. An entinostat (ENT) and BSM-1 co-loaded micelle coated with dextran sulfate (DXS) was prepared. ENT acted as an MDSC inhibitor, BSM-1 was an ICI, and DXS could reshape M2-TAMs to a proinflammatory M1 phenotype by blocking scavenger receptor A 258. Another ROS scavenger, a Zr-CeO nanozyme, was reported to dampen MDSCs by downregulating the unfolded protein response (UPR) and restraining the M2 polarization of TAMs *via* hindering the ERK and STAT3 pathways. This nanozyme boosted the therapeutic efficacy of PD-1 inhibition in both renal and breast cancer 259.

### 3.4. Modulating the metabolism of tumor cells

**Figure 8:**
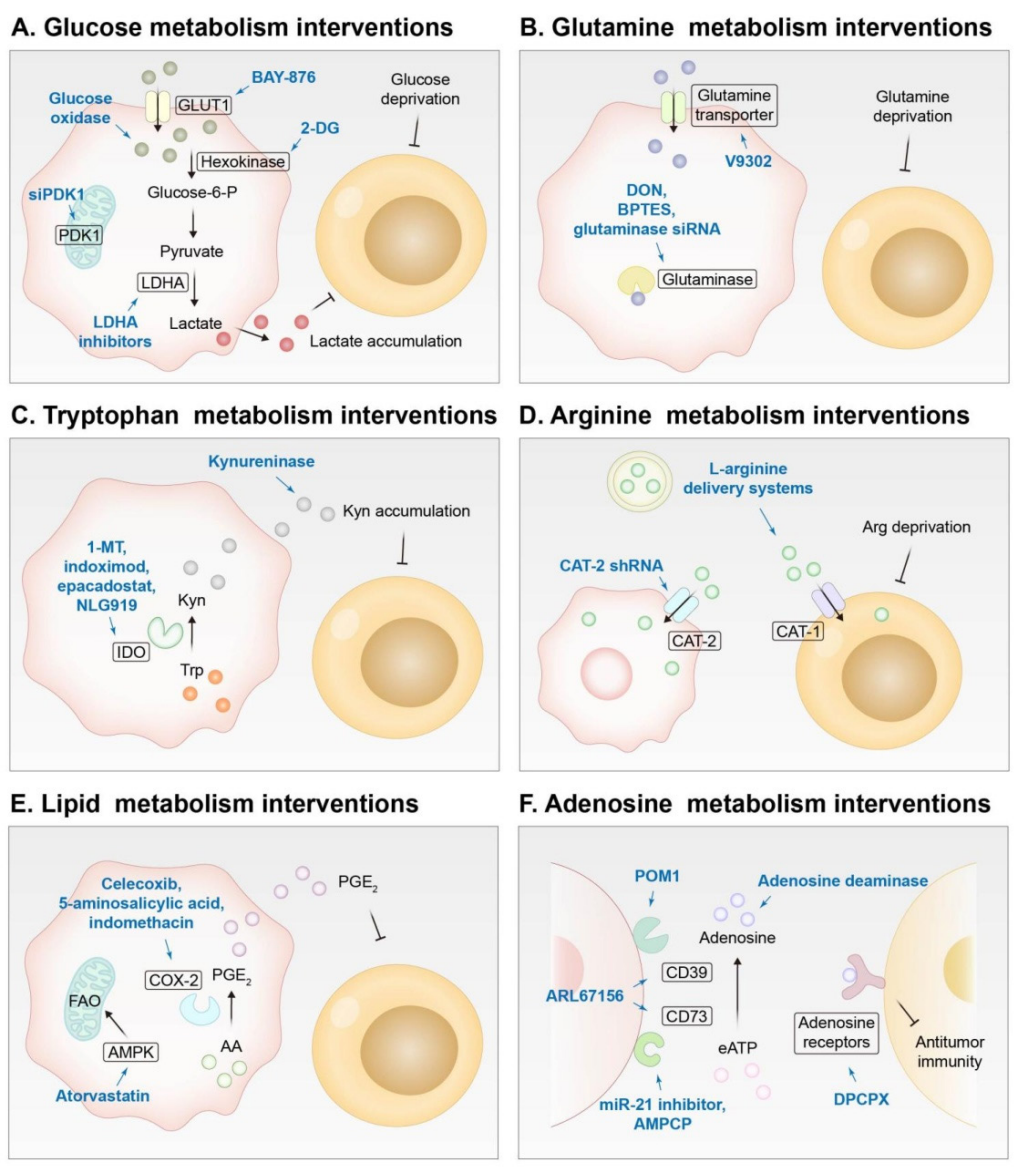
** Improving the efficiency of ICB by regulating the metabolism of cells in the TME using drugs delivered by NDDSs.** Pink for tumor cells, and yellow for T cells. (A) Intervening glucose metabolism of tumor cells by GLUT1 inhibitors, hexokinase inhibitors, glucose oxidase, PDK1 inhibitors, or LDHA inhibitors. (B) Intervening glutamine metabolism of tumor cells by glutamine transporter inhibitors or glutaminase inhibitors. (C) Intervening tryptophan metabolism of tumor cells by IDO inhibitors or kynureninase. (D) Intervening arginine metabolism of tumor cells by CAT-2 inhibition and supplying arginine to T cells *via* L-arginine delivery systems. (E) Intervening lipid metabolism of tumor cells by COX-2 inhibitors or FAO enhancers. (F) Intervening adenosine metabolism of tumor cells by adenosine deaminase and CD39 and CD73 inhibitors or blocking adenosine receptors on T cells. GLUT1, glucose transporter 1; PDK1, 3-phosphoinositide-dependent protein kinase-1; 2-DG, 2-deoxy-D-glucose; LDHA, lactate dehydrogenase A; DON, 6-Diazo-5-oxo-L-norleucine; Kyn, kynurenine; Trp, tryptophan; IDO, indoleamine 2,3-dioxygenase; 1-MT, as 1-methyl-DL-tryptophan; CAT, cationic amino acid transporter; COX-2, cyclooxygenase-2; AA, arachidonic acid; PGE_2_, prostaglandin E_2_; FAO, fatty acid oxidation; eATP, extracellular ATP; POM1, sodium polyoxotungstate; AMPCP, α, β-methylene adenosine 5′ diphosphate; DPCPX, 8-Cyclopentyl-1, 3-dipropylxanthine.

Metabolic reprogramming represents a hallmark of tumors. Rapidly proliferating tumor cells consume a large amount of nutrients, such as glucose and glutamine, which leads to nutrient depletion for immune cells in the TME and aggravation of immunosuppression of the TME. In addition, abnormal metabolism of amino acids, lipids, and nucleotides contributes to the formation of a suppressive immune microenvironment in tumor tissues 260. Recently, an array of nanomedicines have been designed to sensitize ICB by regulating tumor metabolism, and the strategies are summarized in **Figure 8** and will be discussed in this section.

As a primary energy supply mode in cancer cells, glycolysis plays a crucial part in maintaining the energy supply of neoplastic cells, leading to the deprivation of glucose in the TME and the creation of an acidic immunosuppressive TME 261. Inhibition of glycolysis has been shown to enhance ICB potency by restoring glucose supply to immune cells and reshaping the acidic immunosuppressive TME. A nano-assembly derived from poly β-amino ester (PAE) was prepared. This nano-assembly was loaded with BAY-876, a GLUT1 inhibitor, and the PAE moiety was conjugated with PD-L1 and CTLA-4-antagonizing aptamers, which could be released when the PAE moieties were protonated and transformed from hydrophobic to hydrophilic in an acidic TME 38. In another study, siRNA targeting 3-phosphoinositide-dependent protein kinase-1 (PDK1) was delivered to interfere with glycolysis, thus achieving metabolic reprogramming and metastasis modulation 262. Amphiphilic dendrimers were employed as vectors for siPDK1, and PD-L1 antibodies were decorated on the hydrophilic terminal of the dendrimers for targeting tumors and performing ICB. 2-deoxy-D-glucose (2-DG), an antiglycolytic agent, was also reported to be incorporated into nanoplatforms for combination with ICIs 263, 264. A synergetic metabolic nanoregulator was designed by enveloping 2-DG, BAY-876, and chloroquine (an autophagy inhibitor) into ZIF-8 to boost aCTLA-4 immunotherapy 265. A nanosonosensitizer modified with a metabolic regulation peptide R7, which restrained glycolysis of tumor cells but exerted negligible effects on normal cells, was proposed to synergize with aPD-L1 for treating challenging spinal metastasized and distant tumors 266. In addition, due to the positive effect of OXPHOS on antitumor immunity, mPEG-PLA-PHis-ss-PEI polyplexes were developed to co-deliver siPD-L1 and resveratrol, which upregulated OXPHOS and suppressed glycolysis 267. Nanoengineered glucose oxidase has also been reported to enhance the antitumor effect of PD-1/PD-L1 blockade by regulating glucose metabolism 268-271. Moreover, CRISPR/Cas9 and siRNA targeting lactate dehydrogenase A (LDHA) and inhibitors of LDHA such as GSK2837808A were delivered by NDDSs to downregulate lactate production for sensitizing checkpoint blockade 272-274.

Similar to glucose, glutamine in the TME is also overconsumed by tumor cells, which severely weakens the activation of immune cells and impairs their function 275. For example, PLL-modified lamellar molybdenum disulfide (MoS_2_) was constructed for co-delivering aPD-L1 and V9302, an inhibitor of the glutamine transporter that blocks glutamine internalization by tumor cells but not T cells 276. Moreover, V9302 could drive the distribution of tumor-infiltrating lymphocytes from the periphery to the core of carcinoma tissues. This nanosystem significantly increased the glutamine concentration in the TME and evoked a potent antitumor immune response in TNBC. In another report, V9302 blocked GSH synthesis by hindering glutamine uptake and synergized with an HDAC inhibitor MS-275 that promoted the generation of a ROS storm, leading to pyroptosis of tumor cells and boosting aPD-1 efficacy 277. Glutamine antagonists can also be used to reshape the metabolism of tumors and immune cells to benefit their combination therapy with ICIs 278, 279. It is worth mentioning that inhibition of glutamine uptake may lead to a switch of compensatory glycolysis, which exacerbates lactate accumulation, deteriorates the immunosuppressive TME, and even enhances PD-L1 expression on tumor cells. In this context, a PD-L1-targeting metabolism and immune regulator (PMIR) was prepared. It was derived from a glutaminase inhibitor (BPTES)-loaded ZIF that was encapsulated by a liposome and modified with a PD-L1-targeting peptide on the surface 280. ZIF not only acted as a carrier for BPTES, but also released Zn^2+^ in the tumor cells that could reduce NAD^+^ and inhibit glycolysis to counteract glycolytic compensation induced by glutamine metabolism inhibition. In addition, a mesoporous silica nanoplatform was developed to co-encapsulate lonidamine and siRNA for antiglutaminase for combating anti-PD-1 resistant tumors 281.

Tryptophan (Trp) in tumor cells can be catabolized to kynurenine (Kyn) by upregulated indoleamine 2,3-dioxygenase (IDO). The accumulation of Kyn contributes to the loss of the tumoricidal function of T cells and NK cells and the immune escape of cancer cells 282. Nanoparticles, nanosheets, nanovesicles, and liposomes have been exploited to deliver IDO inhibitors, such as 1-methyl-DL-tryptophan, indoximod, epacadostat, and NLG919, for combination therapy of IDO inhibition and checkpoint blockade 283-292. NLG919 and OTX015 (a bromodomain extra-terminal inhibitor) were co-loaded into mesoporous polydopamine nanoparticles for synergistic therapy of IDO inhibition, PTT, and dual immune checkpoints (PD-L1 and CD47) blockade 293. Recently, porous silica nanoparticles (PSNs) were employed to encapsulate kynureninase (KYNase), which hydrolyzed Kyn into anthranilic acid and alanine. By reducing the level of Kyn in the TME, tumor immunosuppression was alleviated, and the therapeutic effect of aPD-1 was improved in CT26, 4T1, and B16F10-bearing mice 294.

L-arginine is essential for the proliferation, differentiation, activation, and function of T cells 295, but its delivery to a tumor site is still challenging due to its hydrophilicity. Fortunately, L-arginine was reported to be decorated with terephthalaldehyde (Ter) *via* an acid-responsive imine bond to allow the formation of ArgNPs (~104 nm) *via* a self-assembly process and acid-triggered release of L-arginine in the TME. ArgNPs, in combination with aPD-L1, dramatically increased the number of tumor-infiltrating T cells and induced the formation of memory T cells 296. Moreover, considering that L-arginine in the TME could promote cancer proliferation when internalized by tumor cells, L-arginine-loaded multivesicular liposomes (L-arg@MVLs) were developed to simultaneously achieve L-arginine supplementation to immune cells and deprivation in tumor cells 297. The authors employed shRNA to downregulate cationic amino acid transporter-2 (CAT-2), which is involved in transmembrane transport of L-arginine into tumor cells, while this shRNA did not impact amino acid transporter-1 (CAT-1) on the surface of CD8^+^ T cells and macrophages. L-arg@MVLs provided the supply of L-arginine to immune cells, and they also neutralized the acidic TME as an alkali, which effectively inhibited the growth of B16 melanoma in the mice when combined with aPD-1.

Lipid metabolic dysregulation contributes to the proliferation of cancer cells and the creation of an immunosuppressive TME 298. Arachidonic acid (AA), an omega-6 (ω-6) polyunsaturated fatty acid, can be catalyzed into prostaglandin E_2_ (PGE_2_), a protumor inflammation mediator, by overexpressed COX-2 in neoplastic tissues 299. Therefore, COX-2 inhibitors have been used to block PGE_2_ production to mitigate immunosuppression of the TME. In combination with antibodies against PD-1/PD-L1, bionic nanoparticles or polymer micelles have been employed to deliver celecoxib, 5-aminosalicylic acid, or indomethacin for COX-2 inhibition, thus reducing the presence of immunosuppressive MDSCs and TAMs while enhancing the antitumor activity of Teffs 115, 300, 301. In addition, fatty acid oxidation (FAO) is usually downregulated in tumor cells to avoid oxidative damage caused by excess ROS production. A lipopeptide nanoplex loaded with atorvastatin (ATO) and siPD-L1 was reported to treat melanoma and colorectal cancer. Adenosine monophosphate (AMP)-activated protein kinase (AMPK) stimulated by ATO restored the downregulated FAO and promoted ROS production, and continuous production of ROS led to tumor cell killing. Moreover, ATO could inhibit triglyceride synthesis to ensure intracellular fatty acid availability. The ICD effect caused by excess ROS combined with PD-L1 silencing resulted in a favorable antitumor efficacy 302.

In addition, nucleotide metabolism may influence the formation of an immunosuppressive TME. Under the action of overexpressed ectonucleotidases, including CD39 and CD73, extracellular ATP in the TME can be converted into adenosine, which promotes the infiltration of immunosuppressive cells and inhibits the proliferation and impairs the function of immune effector cells by binding to adenosine receptors 303. Hence, inhibiting ectonucleotidases and eliminating adenosine in the TME, or blocking adenosine receptors on immune effector cells, have been studied to alleviate tumor immunosuppression and enhance the effect of ICIs. Glioma-associated mesenchymal stem cells (GA-MSCs) were revealed to promote CD73 expression on MDSCs *via* exosomal miR-21 signaling. DC-derived exosomes were decorated with angiopep-2, a BBB-penetrating peptide, for GA-MSC-targeting delivery of a miR-21 inhibitor. The resulting nanomedicine, Dex-miR-21 inhibitor, downregulated CD73 expression on MDSCs. The combination of the Dex-miR-21 inhibitor and aPD-1 significantly extended the survival of GL261 glioma-bearing mice 304. Besides, sodium polyoxotungstate (POM1, a CD39 inhibitor) 305, α, β-methylene adenosine 5′ diphosphate (AMPCP, a CD73 inhibitor) 306, 307, and ARL67156 (a dual CD39/CD73 ectonucleotidase inhibitor) 308 have been employed to restrain the ectonucleotidases activity for sensitizing ICB. In another report, 8-cyclopentyl-1, 3-dipropylxanthine (DPCPX), an adenosine A1 receptor inhibitor, and anti-PD-L1 DNAzyme were co-delivered by a core-shell nanoparticle for melanoma treatment 309. Moreover, a nano-immunocomplex comprised of adenosine deaminase, a sonosensitizer, and aPD-L1 was designed for sono-metabolic trimodal cancer therapy, which exhibited a distinguished antitumor effect in 4T1-bearing mice 310.

### 3.5. Remodeling non-immune components in the TME

**Figure 9:**
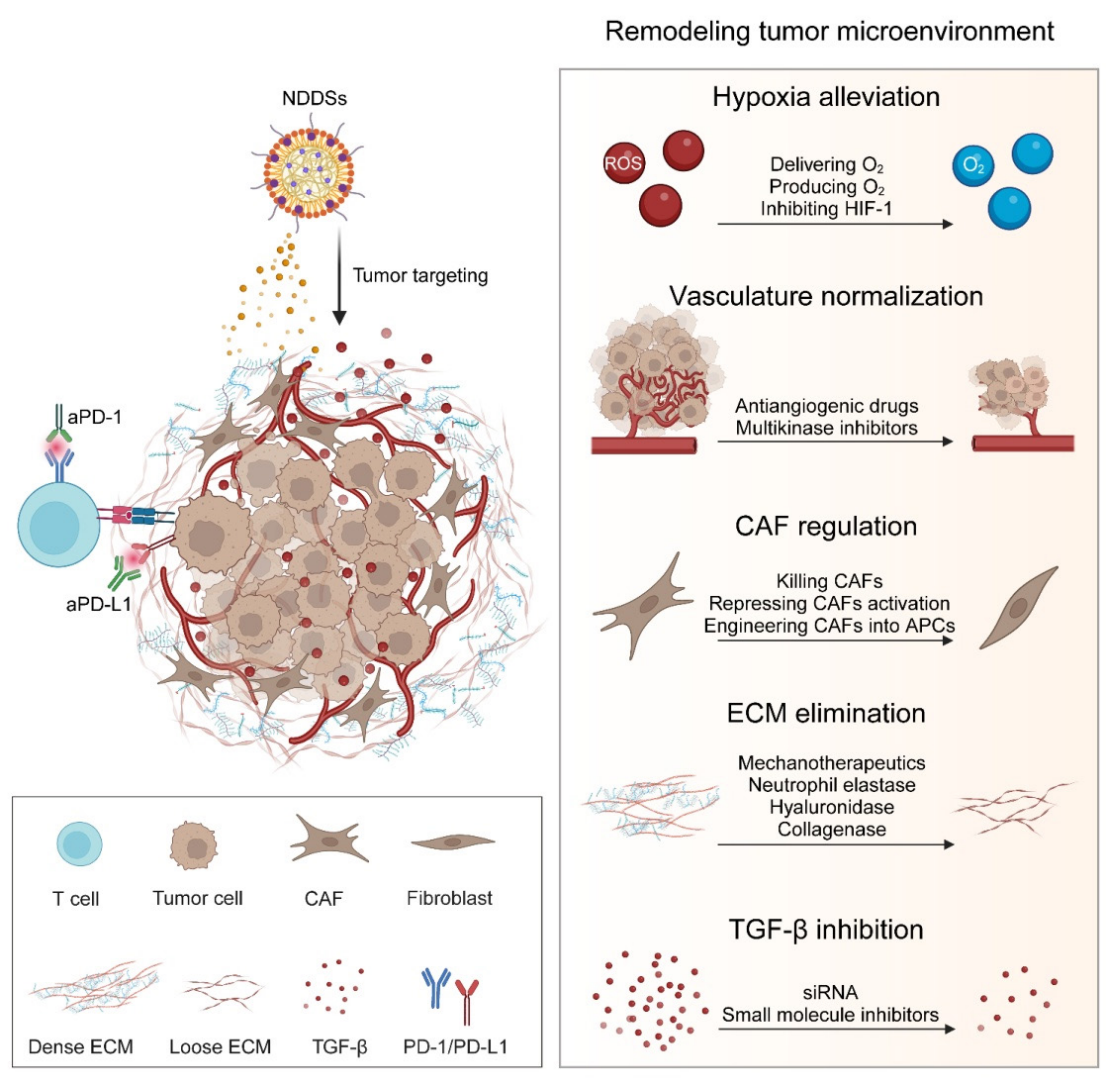
** Strategies of reprograming non-immune components in the TME for superior antitumor effects of ICIs include alleviating hypoxia by delivering or producing O_2_ or inhibiting HIF-1, normalizing vasculature by antiangiogenic drugs or multikinase inhibitors, regulating CAFs by killing CAFs, suppressing CAF activation or engineering CAFs into APCs, eliminating ECM by methanotherapeutics, neutrophil elastase, hyaluronidase or collagenase, and inhibiting TGF-β using siRNA or small molecule inhibitors.** HIF-1, hypoxia inducible factor-1; CAF, cancer-associated fibroblast.

In addition to immune cells, non-immune stromal components in the TME, including stromal cells, ECM, vasculature, and soluble factors, interact with tumor and immune cells synchronously, and they play a pivotal regulatory role in cancer progression and immune response 311, 312. These components have been targeted to enhance the efficacy of ICIs. The reported NDDSs for targeting non-immune stromal constituents in the TME to sensitize ICB are elaborated in **Figure 9**.

Hypoxia is one of the prominent features of the TME, and it results from excessive oxygen consumption by rapidly proliferating tumor cells and abnormal angiogenesis in the neoplasm. Hypoxia and aberrant vasculature not only seriously hinder the penetration of drugs and diminish their efficacy but also strengthen immune suppression of the TME 313. Hence, various strategies have been formulated to alleviate tumor hypoxia. Three hypoxia alleviation strategies, including liposomal perfluorocarbon (PFC@lipo), liposomal hemoglobin (Hb@lipo), and PX-478, a commercial inhibitor for hypoxia-inducible factor-1α (HIF-1α, a hypoxia-activated transcription factor that encodes a variety of proteins responsible for cancer progression), have been compared and Hb@lipo has been identified to possess the best tolerance and safety and improve the effect of aPD-1 314. Interestingly, photosynthesis microcapsules were constructed with acquired cyanobacteria and upconversion nanoparticles, in which long-lasting oxygen supply at a tumor site could provide an oxygen-rich microenvironment, resulting in an enhancement of synergistic therapeutic effect with aPD-1 315. More recently, oxygen-encapsulated polydopamine nanoparticles were also synthesized to enhance the efficacy of aPD-1 316. Moreover, hypoxia is the culprit of inefficacious PDT and resistance to chemotherapy and radiotherapy. MnO_2_ nanoparticles have exhibited a higher catalase-like activity than catalase and other nanozymes. MnO_2_ has been utilized to mitigate hypoxia and boost combination immunotherapeutic effects with ICB 317-322. For example, ICG-loaded hollow MnO_2_ nanospheres coated with aPD-L1-modified exosomes were prepared. They bolstered the efficacy of PDT and ICB by elevating the O_2_ level in the TME of non-small cell lung cancer (NSCLC) 317. In addition, other nanoplatform-based agents to alleviate hypoxia in combination with ICIs include platinum nanoparticles 323, 324, metformin 325, 326, and Prussian blue 327. Anti-angiogenesis represents another strategy to cope with hypoxia and enhance immunotherapy 328. Apatinib 329, sorafenib 330, DC101 331, and antiangiogenic peptides 332 have been incorporated into NDDSs for combined treatment with ICB.

Many types of cancer, especially pancreatic and breast cancer, are characterized by dense stroma, which contributes to tumor stiffness, a high interstitial fluid pressure (IFP), compressed vessels, and low perfusion, resulting in poor penetration of therapeutic agents and deficient infiltration of immune cells 333, 334. Therefore, dense stroma often accounts for the dismal efficacy of ICIs in desmoplastic tumors. Blocking the production and deposition of stroma appears as an appealing approach to sensitizing ICIs. CAFs are activated and stimulated by TGF-β in tumor tissues. They not only secrete abundant extracellular matrix proteins but also attract suppressive immune cells and repel CD8^+^ T cells 335. Hence, targeting CAFs could facilitate the delivery of immunotherapeutic agents and the infiltration of cytotoxic T lymphocytes for cancer immunotherapy. An oxymatrine (Om) and astragaloside IV (As) co-loaded MOF-based nanoparticle camouflaged with the platelet membrane was reported to suppress the activation of CAFs and improve the activity of tumor-infiltrating T lymphocytes (TILs) by promoting the mitochondrial function, which boosted the antitumor efficacy of aPD-L1 336. Dendritic poly[OEGMA-Dendron(G2)-Gly-Phe-Leu-Gly-DAS] (P-DAS, a prodrug of dasatinib) was synthesized to reprogram CAFs for reducing collagen anabolism and energy metabolism, which led to deeper penetration and superior efficacy of poly[OEGMA-Dendron(G2)- hydrazone-Epi] (P-Epi, a prodrug of epirubicin) and aPD-1 337. In another study, a molecular pro-theranostic probe was employed to simultaneously kill CAFs and cancer cells 338. Melittin was also reported to induce ICD and transform activated CAFs into quiescent cells, and it collaborated with nitric oxide (NO) to enhance the potency of aCTLA-4 339. In addition, inspired by similar characteristics of CAFs as APCs, a photoactivatable thermochromic gene expression nanoplatform was developed by assembling a molten eutectic mixture, chitosan, and a fusion plasmid encoding CD86 and a PD-L1 trap. This nanoplatform was demonstrated to convert CAFs into APCs and block PD-1/PD-L1 signaling. The expression of CD86, a co-stimulatory molecule, resulted in the conversion of CAFs ("foe") to engineered CAFs ("friend") for processing and presenting tumor-associated antigens, which dramatically suppressed the progression of highly fibrotic 4T1 breast cancer in conjunction with PD-L1 blockade 340.

**Figure 10:**
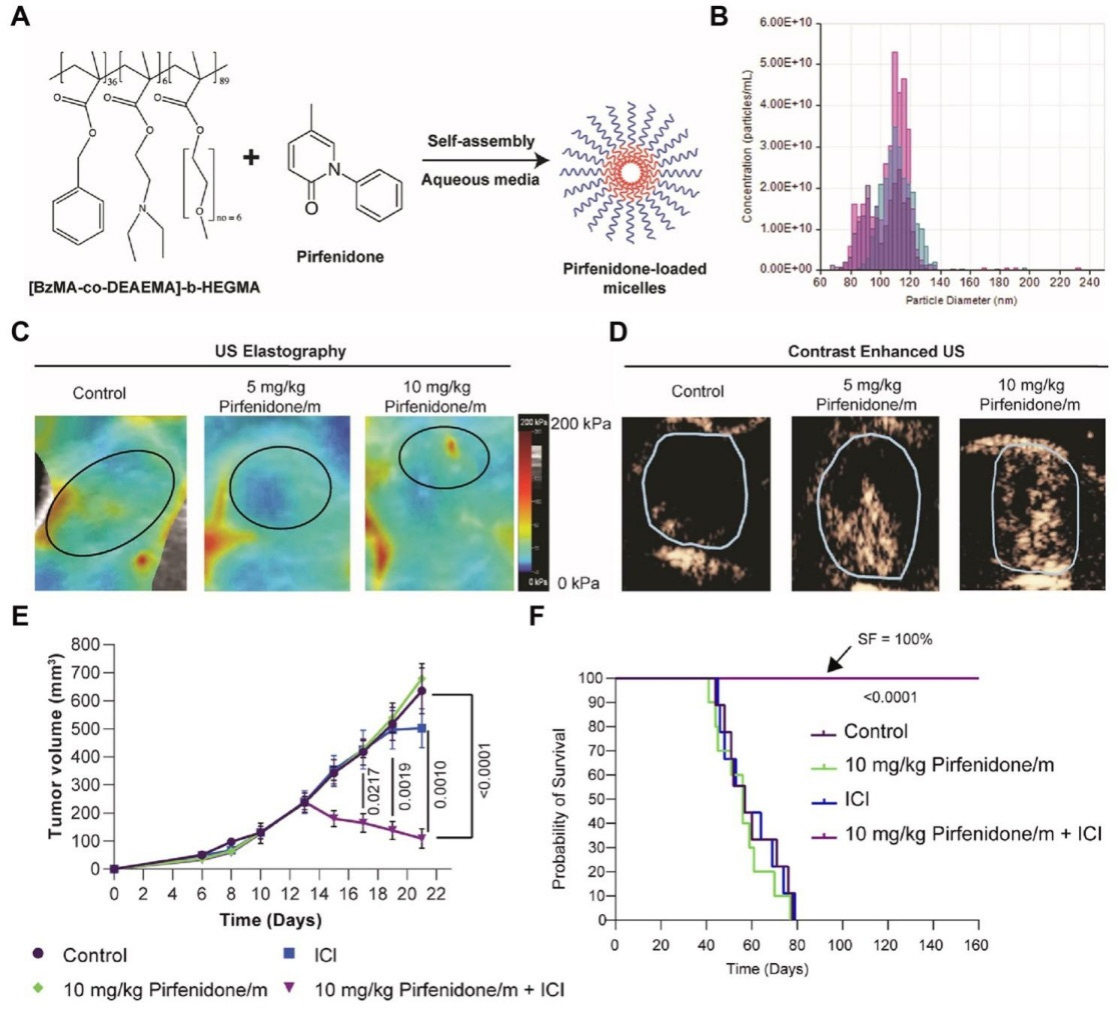
** Mechanotherapeutics delivered by an NDDS for enhancing the efficacy of ICIs by reducing the stiffness of tumor stroma, decreasing the IFP, and increasing drug perfusion.** (A) Schematic of the formation of a pirfenidone-loaded micelle (Pirfenidone/m). (B) Size distribution of the micelles. Representative images of (C) shear wave elastography (SWE) and (D) contrast enhanced ultrasound (CEUS) to support a significantly reduced stiffness and an increased perfusion rate after treatment with Pirfenidone/m. The lines delineate the tumor margins. (E) Growth curves of E0771 tumors (n = 9 or 10 mice). (F) Kaplan-Meier survival curves of the E0771 tumor model after various treatments. Reproduced with permission from ref 342. Copyright 2023, American Chemical Society.

Mechanotherapeutics, such as antihypertensives, antihistamines, antifibrotic agents, and corticosteroids, have been demonstrated to normalize the TME components. Mechanotherapeutics-containing NDDSs for delivering chemotherapeutic or immunotherapeutic drugs could target specific extracellular matrix components or CAFs to remodel the TME 341. Meanwhile, NDDSs can be designed to tailor the dose requirement for mechanotherapeutics, broaden their treatment window, augment their tumor accumulation, and relieve their side effects beyond TME normalization when administrated systemically. A micelle of [BzMA-co-DEAEMA]-b-HEGMA was prepared, and pirfenidone, an antifibrotic drug, was encapsulated into the micelle. The application of the pirfenidone-containing micelle (Pirfenidone/m) reduced the hyaluronan and collagen content in the TME at a pirfenidone dose 100-fold lower than that of its free form, resulting in a reduction in the stiffness of the stroma and the IFP within the tumor tissue, but an enhancement in the accumulation and penetration of delivered ICIs at the tumor site, thereby significantly improving the therapeutic effect of aPD-1+aCTLA-4 **(Figure 10)** 342. In addition, neutrophil elastase-decorated biomimetic liposomes were prepared to degrade elastin and type I collagen in the TME and improve the accumulation of PTX and aPD-1 at the tumor site 343. In another study, CPT prodrug-derived nanofibers were constructed to co-deliver two DNA plasmids expressing shRNA of PD-L1 (shPD-L1) and hyaluronidase (HAase). The fiberlike morphology of the nanocarrier and HA degradation by HAase contributed to enhanced penetration of shPD-L1 and boosted its efficacy 344. Furthermore, multi-targeted tyrosine kinase inhibitors can be used to remediate collagen deposition and abnormal vasculature for effective synergetic therapy with ICIs 345-347.

In addition to the type I collagen (Col1) heterotrimer (α1/α2/α1) secreted by fibroblasts, the oncogenic collagen, the Col1 homotrimer (α1/α1/α1), has been revealed to play a role in the formation of an immunosuppressive TME in pancreatic ductal adenocarcinoma (PDAC). The Col1 homotrimer is induced in cancer cells due to epigenetic silencing of the COL1A2 gene, and it upregulates the FAK/EGFR/MAPK/MYC signaling network by interacting with integrin α3β1, promoting the proliferation of neoplastic cells and development of their therapeutic resistance as well as the recruitment of immunosuppressive cells. A ROS-sensitive protein cage was designed to accommodate collagenase and aPD-L1, and the cage was attached to the surface of a probiotic *Escherichia coli* strain Nissle 1917 (ECN). The hypoxia tropism of ECN and its outstanding motility facilitated efficient penetration of collagenase into deep tumor tissues, where the oncogenic collagen is deposited. This nanodrug-bacteria conjugate markedly strengthened the immunotherapeutic effect against PDAC 348.

Notably, TGF-β is a prominent immunosuppressive cytokine secreted by neoplastic cells, CAFs, and TAMs, and it plays a pivotal role in angiogenesis, ECM deposition, and immunosuppression in the TME 349, 350. Therefore, enhancing the therapeutic effect of ICIs by targeting TGF-β has been extensively explored. For example, an HAase and GSH dual-responsive nanosystem with a coating layer of hyaluronic acid (HA) was constructed to deliver TGF-β siRNA to tumor cells and CAFs, which inhibited both primary and distant tumors when combined with aPD-L1 in a 4T1 breast cancer model 351. In addition, a multifunctional nanodrug was reported to exert a significantly synergetic effect with aPD-L1. In this nanodrug, salvianolic acid B was exploited to inhibit TGF-β signaling and upregulate PD-L1 expression, and a photothermal agent was used to trigger ICD 352. Furthermore, small-molecular inhibitors, such as galunisertib 353, 354, SB-505124 355, 356, LY3200882 357, and LY2157299 358, have been utilized to block the TGF-β signal pathway for improving ICB-based immunotherapeutic effects.

### 3.6. Multifunctional immunoregulatory NDDSs

Immune system activation is a multifaceted process involving several steps and multiple factors. Manipulating a solitary factor may provide inadequate immune response activation, resulting in immune evasion and resistance to ICB. NDDSs with multiple modulations can theoretically activate effective immune responses and achieve a synergistic antitumor effect. These nanomedicines have the capacity to enhance immunogenicity, regulate the metabolism of both tumor cells and immune cells, activate immune cells, and reshape the tumor microenvironment (TME). For example, a biomimetic nanoplatform (SS@Lipo/Hb/GOx/JQ1) was constructed by modifying a Hb, GOx, and JQ1 co-loaded liposome with spore shells (SSs) of *Bacillus coagulans*. In this system, SSs with an adjuvant activity could activate immune cells, and GOx catalyzed glucose into ROS, which triggered ICD under the action of Fe^2+^ in Hb. Meanwhile, Hb relieved hypoxia in the TME, and JQ1 downregulated the expression of PD-L1 359. A radio-immunostimulant nanomedicine (IPI549@HMP) was engineered, in which hollow MnO_2_ was employed as a drug delivery carrier and a catalyst of hydrolyzing H_2_O_2_ to alleviate hypoxia and potentiate radiotherapy, and IPI549 was applied to eliminate immunosuppressive myeloid cells. IPI549@HMP-mediated radiotherapy in synergy with aPD-L1 could establish immune memory to combat tumor rechallenge, leading to inhibition of residual and distant tumors 360. In another study, a multifunctional nanosystem composed of CpG ODNs, an immunostimulating adjuvant, and siRNA cocktails, including STAT3 siRNA to induce ICD, CCR2 siRNA to trigger MDSCs, and TGF-β siRNA to alleviate immune suppression was designed. Moreover, cholesterol-modified AMP DP7 (DP7-C) with an adjuvant activity was exploited as the nanocarrier for the multifunctional nanosystem. The constructed DP7-C/siRNAs/CpG ODNs resulted in a pronounced antitumor effect in both CT26 and B16F10 tumor models when combined with aPD-1 361. Many studies have shown that multifunctional immunoregulatory NDDSs can exhibit excellent antitumor efficacy, generate abscopal effects to prevent distant metastasis, and provide long-term immune memory against tumor recurrence.

Multifunctional NDDSs could also be employed for integrated theranostic design. The activation status of the immune system can be assessed through non-invasive visualization of immune responses *via* an imaging probe in an NDDS, and ICB can be implemented by delivering ICIs through the NDDS in a controllable manner. In this context, precise imaging-guided ICB could be achieved 362. Conventional clinical detection methods, such as ultrasound, PET/SPECT, MRI, and CT, can be employed to monitor immune responses 363, 364. An array of NDDS platforms have been utilized for tumor imaging, for example, microbubbles for ultrasound imaging, liposomes, dendritic macromolecules, or polymer nanoparticles loaded with MRI contrast agents (e.g., iron oxide, gadolinium ions, or manganese ions), gold nanoparticles as inorganic carriers for CT imaging, and tracers containing radioactive isotopes or fluorescent groups for PET and SPECT 365, 366. In order to enable real-time and dynamic monitoring, NDDSs are often armed with TME stimuli-responsive groups. Optical imaging is the most extensively studied method, particularly when activatable molecular probes are used. Biomarkers associated with immune activation can be harnessed for the development of optical imaging methods 367, 368. These probes generate signals exclusively in the presence of specific biomarkers (e.g., PD-L1/PD-1, granzyme B, and ROS), thereby significantly enhancing detection specificity and minimizing background noises. This technique allows non-invasive, real-time visualization of imaging probes distributed in the body and biomarkers that are targeted by these imaging probes. Other imaging strategies have also made progress. Silica-coated iron oxide nanoparticles for MRI and micro-computed tomography (μCT) imaging were employed for non-invasive diagnosis of solid tumors. Additionally, a system responsive to MMP-2 in the tumor microenvironment to realize controlled release of drugs was developed for targeted delivery of PD-L1 inhibitors 369. 68Ga-NOTA-Nb109, a single-domain antibody with a special structure and a small molecular weight, could preferably accumulate into the A375-human PD-L1 tumor and could realize non-invasive and dynamic imaging of PD-L1 *in vivo* 370. Similar nanoprobes labeled with other contrasts have been constructed to target PD-L1 371.

In summary, NDDSs with multifunctional immune-modulating ability can improve the permeability and distribution of therapeutic agents in tumor tissues and have been widely applied in combination immunotherapy. NDDSs can be constructed with therapeutic and diagnostic agents. They could also provide real-time monitoring of immunotherapy efficacy, reducing irAEs and resistance to ICB. This approach demonstrates great potential in enhancing cancer theranostics and promoting treatment effectiveness.

## 4. Emerging Strategies for ICB

In addition to conventional ICIs, emerging ICB strategies based on NDDSs to induce checkpoint protein degradation or indirectly inhibit checkpoint expression using therapeutic drugs have been extensively explored. Instead of using antibodies, nucleic acids, or small molecular ICIs, the ICP pathway can be effectively blocked by targeting the degradation of PD-L1, inhibiting PD-L1 palmitoylation, activating AMPK by suppressing OXPHOS, alleviating hypoxia or other mechanisms. These emerging strategies could open a new avenue for ICB.

**Figure 11:**
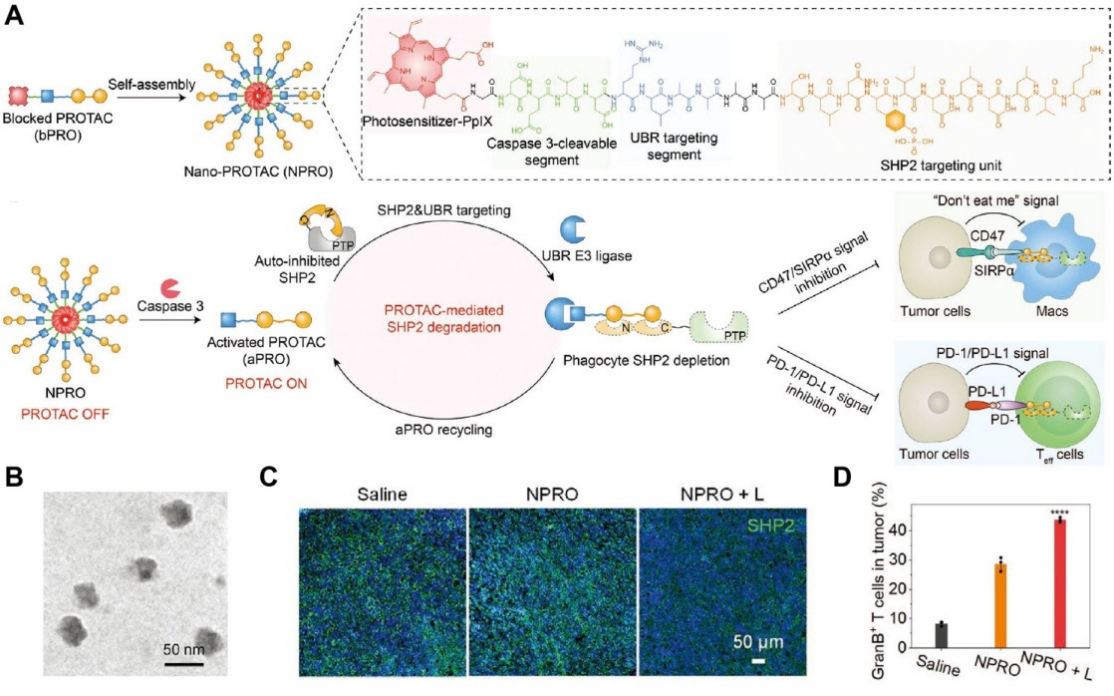
**NDDS-based proteolysis targeting chimera (PROTAC) for immune checkpoint blockade.** (A) Construction of a checkpoint nano-PROTAC (NPRO) and its chemical structure. Schematic illustration of NPRO-mediated cancer photo-immunotherapy: degradation of SHP2 by caspase 3-specifically activated NPRO to inhibit CD47/SIRPα signaling in macrophages (Macs) and the PD-1/PD-L1 axis in T cells, leading to enhanced phagocytosis effects and antitumor T-cell immunity. (B) TEM image of NPRO. (C) SHP2 immunofluorescence staining images and MFIs of 4T1 tumor tissues after treatment with saline, NPRO, and NPRO + L. Blue for DAPI and green for SHP2. (D) GranB^+^CD8^+^ T cells in 4T1 tumors after different treatments (n = 3). Reproduced with permission from ref 372. Copyright 2023, Wiley.

Proteolysis targeting chimera (PROTAC), a protein degradation technology, has been employed in disease treatment by inducing targeted protein degradation *via* the ubiquitin-proteasome system (UPS). Recently, a checkpoint nano-PROTAC (NPRO) for blocking CD47/SIRPα and PD-1/PD-L1 by targeting the Src homology 2 domain-containing phosphatase 2 (SHP2) was reported 372. The checkpoint NPRO self-assembled from the blocked PROTAC peptide (bPRO) composed of protoporphyrin IX, a caspase 3-cleavable segment (DEVD), a UBR E3 ligase-targeting unit, and a SHP2-targeting peptide. Upon ICD induced by PDT, DEVD was cleaved by caspase 3, and the activated PROTAC (aPRO) was subsequently released for the depletion of SHP2 **(Figure 11)**. Similarly, a PROTAC of bromodomain and extraterminal protein 4 (BRD4) for epigenetic regulation of PD-L1 and c-Myc 373 and a carbon-dot-based PROTAC of the PD-L1 protein have been developed for cancer immunotherapy 374. PROTAC has been explored for the degradation of pathogenic proteins since the discovery of small molecular inhibitors for these proteins is challenging. It has shown great potential in treating many diseases, including cancer. Complex chemical synthesis steps, risks of immunogenicity and off-target, and high development costs are critical obstacles to future application of the PROTAC technology 375-377.

The palmitoylation of PD-L1 by palmitoyltransferase ZDHHC3 (DHHC3) has been demonstrated to block PD-L1 ubiquitination and degradation, leading to the redistribution of PD-L1 on the plasma membrane and a reduction in therapeutic benefits of ICB 378. Encouraged by this finding, 2-bromopalmitate, a palmitoylated inhibitor, was incorporated into polymer-lipid hybrid nanoparticles for ICB treatment 379. Harnessing superior targeting specificity of competitive peptides, another team designed a nano-inhibitor (FRS) for PD-L1 palmitoylation. The nano-inhibitor was composed of a fluoroalkylated competitive peptide (RMMDVKKCGIQDTNS, termed "RS"). The fluoroalkylated peptide and DOX self-assembled into a nanoparticle 380. After the nanoparticle was transported into tumor cells, the disulfide bond between RS and the fluorous tag could be cleaved to release RS and DOX. RS competed with PD-L1 in palmitoylation by DHHC3. Lysosomal clearance of PD-L1 combined with the released chemotherapeutic DOX triggered a powerful antitumor immune response in a CT26 mouse tumor model.

Elevated glycolysis and OXPHOS levels in tumor cells lead to a decrease in intracellular adenosine diphosphate (ADP)/ATP and AMP/ATP ratios, which impedes the activation of AMPK. Activating AMPK by inhibiting OXPHOS can phosphorylate PD-L1 and promote its ER accumulation and ER-associated protein degradation (ERAD) 381, 382. Inspired by this, a series of nanosystems derived from albumin nanoparticles or liposomes were developed to deliver mitochondria-targeting OXPHOS suppressors 383-387. Mitochondria-targeting triphenylphosphonium cation (TPP^+^) or heptamethine cyanine dyes (MHI or IR-68) have been chemically conjugated with an OXPHOS disruption agent (metformin, lonidamine, or tamoxifen) to improve the OXPHOS inhibition efficiency and reduce the therapeutic dose of OXPHOS suppressors.

It is well known that PD-L1 is a downstream target gene of HIF-1. In this context, suppressing HIF-1 by alleviating hypoxia or applying HIF-1 inhibitors has been widely used to downregulate PD-L1. Hemoglobin 388, catalase 389, hyaluronidase 390, γ-Fe_2_O_3_ 391, manganese oxide nanoparticles 392-394, and mesoporous platinum nanoparticles 395 are reported to decrease PD-L1 by mitigating hypoxia and improve antitumor immune responses. HIF-1 inhibitors, including acriflavine 396 and BAY87-2243 397, are also utilized to curtail the expression of PD-L1 in nanoplatform-based immunotherapy. Moreover, glucosamine-labeled liposomal ceramide was reported to block HIF-1-mediated PD-L1 expression 398. Other strategies for downregulating immune checkpoints are listed in **Table 2**.

## 5. Perspectives and challenges

**Figure 12:**
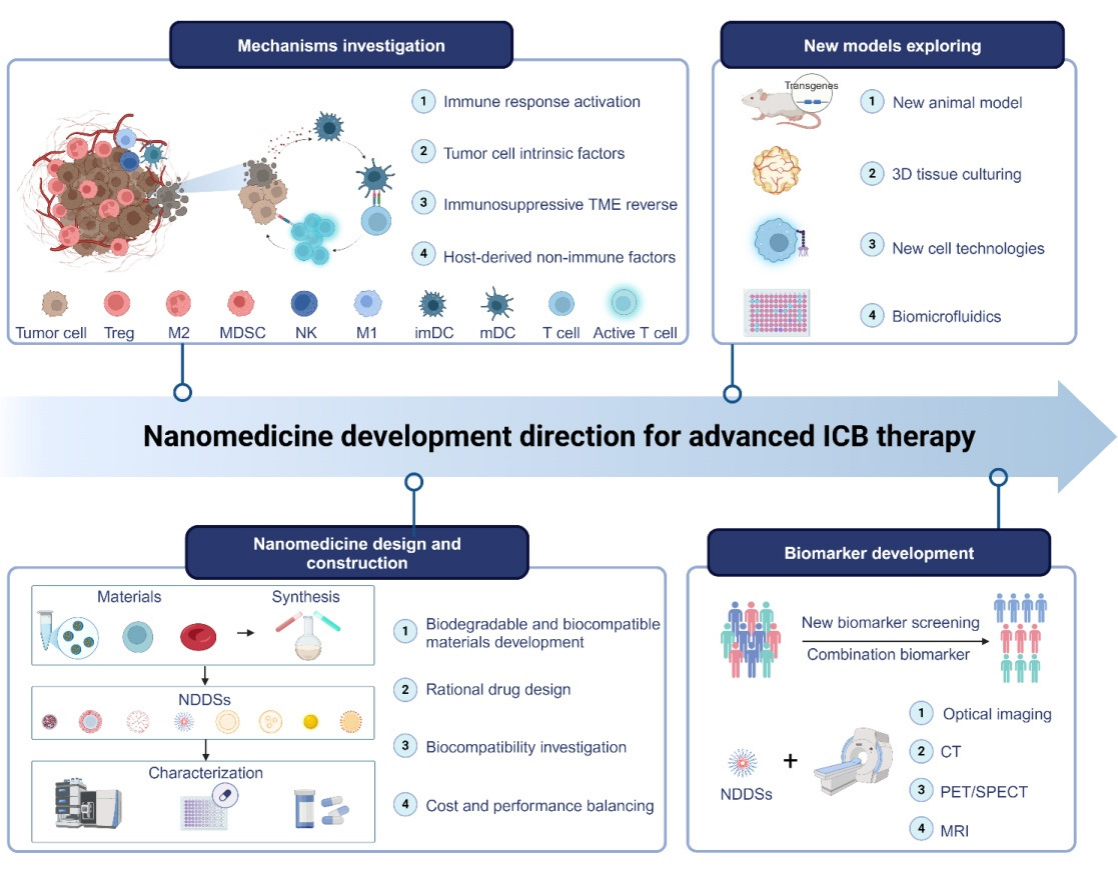
** Nanomedicine development directions for advanced ICB therapy.** CT, computed tomography; PET, positron emission tomography; SPECT, single-photon emission computerized tomography; MRI, magnetic resonance imaging.

Numerous studies have utilized NDDSs to address irAEs and drug resistance associated with ICIs and improve immunotherapeutic efficacy. Strategies have been developed to prolong the drug circulation time, minimize effective doses, improve tumor targeting specificity, reduce non-specific uptake, and promote remodeling of the TME. However, very few NDDS-based therapeutic nanomedicines have been translated into clinical application. Extensive studies on intricate mechanisms of ICI resistance and irAEs, NDDS construction strategies, treatment effect monitoring technologies, and combinational optimization of therapeutic agents are required to successfully translate these NDDS-based therapeutic nanomedicines into clinical trials **(Figure 12)**.

### 5.1. Mechanism investigation

It is crucial to recognize that conventionally used strategies may be insufficient to address complex and multifaceted challenges of response and resistance to ICIs 17. It has been found that the mechanisms of immune escape vary in different types of tumors, and even within a specific tumor type, immune evasion mechanisms can exhibit significant variations 411. Although combinational drugs to achieve direct tumor cell elimination and TME modulation have shown enhanced immunotherapeutic efficacy in certain animal models, the effectiveness of such combinations may be compromised by intricate interactions among drugs, tumor cells, immune cells, TME components, and signaling/chemotactic factors 412. Therefore, an in-depth revelation of these interactions and the underlying mechanisms of irAEs and ICI resistance can tailor immunomodulation strategies and facilitate optimal antitumor efficacy.

The underlying mechanisms of ICI resistance are related to tumor cells, their TME, and the host. (1) Tumors can inhibit the immune response by downregulating presentation antigens, suppressing APC functions, increasing inflammatory cytokine accumulation, and restraining T cell activation and infiltration. The processes by which cancer neoantigens are internalized by APCs and subsequently cross-presented to naive CD8^+^ T cells, and the factors that modulate the activation and function of both naive and primed CD8^+^ T cells are critical for eliciting effective antitumor immune responses; (2) Tumor cells may adapt compensatory metabolic pathways upon introducing metabolic interventions, leading to acquired resistance 65. Identifying key metabolic pathways in different types of cancer cells could facilitate the development of specific and effective metabolic intervention strategies to suppress tumor proliferation without inducing resistance. Since tumor cells in different regions of a tumor site exhibit distinct metabolic characteristics, which may dynamically change with tumor progression, metabolic intervention strategies must account for the spatial-temporal heterogeneity of tumor cells within the TME; (3) It has been shown that only less than 20% of patients exhibit an immune-responsive TME. In contrast, the majority of patients have an immunosuppressive TME, resulting in poor responses and prognoses to ICB therapy. The TME is a complex system constituted of various types of immune cells, stromal cells, dense ECM, and abnormal vasculature. All these components are dynamically altered, interdependently interactive with each other, and synchronously coordinated to establish a favorable environment for tumor progression and metastasis 17, 413. The current understanding of these components, especially interactions between these components, remains the tip of the iceberg, and profiling the TME helps the development of therapeutic drugs to destroy tumor physical barriers, modulate the TME composition, and activate effector T cells in the TME; (4) Host-derived non-immune factors, such as microbiomes, hormones, and neuronal signaling molecules, have been shown to play a role in ICI response and resistance 12, 414. Correlations between the clinical efficacy of ICIs and race, age, gender, lifestyle, diet, and other factors of patients remain to be discovered through advanced data analytics.

### 5.2. Safety issues stemmed from nanomedicine construction

Despite the demonstrated superior efficacy of many nanodrugs, there are challenging issues associated with NDDSs for ICB therapy. To improve the therapeutic efficacy of NDDS-based ICIs and ensure their biosafety, a variety of factors should be taken into consideration for NDDS design and preparation: (1) Material chemistry is the core of NDDSs, and novel biodegradable and biocompatible materials should be developed to meet safety needs for ICB therapy; (2) Different properties of drugs and their dosage ratios should be considered during design and preparation of NDDSs because pharmacokinetic (PK) and pharmacodynamic (PD) performances of these drugs can be pronouncedly improved by NDDSs. Therefore, more studies should be implemented on studying PK/PD of combination treatments with diverse kinds of drugs and investigating their application in different types of tumors; (3) Smart design endows NDDS-derived nanomedicines with the capability of targeting specific tissues, cells, and subcellular organelles, which may dramatically boost therapeutic effects of drugs. For example, stimulus-responsive nanomedicines can release drugs controllably by designing and constructing specific structures to respond to endogenous/exogenous stimuli. Studies should be conducted to investigate the stimuli-responsiveness of these structures and fine-tune the balance between the efficacy and the safety of ICIs; (4) Too complex drug delivery systems may not be conducive to large-scale production, quality control, and clinical translation of nanomedicines. The lack of efficient purification methods, a high cost of large-scale preparation, a low production yield, and challenging issues in quality control have hampered large-scale NDDS production. Collaborative efforts among material chemists, engineers, and clinicians could be essential to overcome obstacles to NDDS production in a good manufacturing practice (GMP) facility.

### 5.3. Biomarker development

Biomarkers could be used to predict/monitor/assess the therapeutic effects of ICB therapy. The discovery of reliable and specific biomarkers is essential for accelerating the transformation of ICB therapy into clinical application. Ideal biomarkers are expected to assist ICB therapy from multiple perspectives, including identifying patients who may positively respond to ICIs, assisting clinicians with decision-making processes *via* prognosis assessment regarding disease progression and patient survival duration, or predicting potential adverse events associated with the therapy *via* evaluating the effects of ICIs on the immune system. To date, PD-L1 and MSI-H/dMMR have been officially approved for clinical use as biomarkers, and they have shown great promise in certain indications 415, 416. Additional biomarker candidates are actively sought. Based on their sources, these biomarkers can be categorized into three types: immune biomarkers, genetic biomarkers, and biomarkers in peripheral blood. Among all these biomarkers, PD-L1 expression, microsatellite instability (MSI), tumor mutation burden (TMB), and mismatch repair deficiency (dMMR) have been well-established to determine whether patients can benefit from ICIs 417, 418. An elevation in the PD-L1 expression is associated with a preferable prognosis 11, 419. A high level of MSI indicates a high mutation burden and great sensitivity to ICIs 420. A greater level of TMB is often correlated with more neoantigens on tumor cells, which could promote attacks by the immune system 421. The majority of these biomarkers are from tumor tissues, which may hamper their application due to the high demand for adequate tissue samples and the invasive nature of the procedure of obtaining tissue samples. Therefore, blood biomarkers are preferable in future clinical applications. Apart from discovering novel biomarkers, strategies for simultaneously harnessing two or more biomarkers have yielded comprehensive benefits. For example, combining TMB with PD-L1 expression exhibited better prediction than one single biomarker 422.

NDDSs have been used to combine ICIs with imaging probes for one or multiple biomarkers, enabling monitoring of immune responses and disease progression by detecting specific immune cell types, metabolites during/after immune activation, or biomarkers for indicating the immune cell status, therefore, precise prediction, real-time monitoring, and accurate assessment of the therapeutic effect of ICIs can be achieved. However, challenges to the clinical translation of NDDS-aided imaging techniques remain in many aspects. Optical imaging, the widely investigated method, has drawbacks, including poor tissue penetration and insufficient depth of examination 367. On the contrary, clinical routine imaging techniques, such as CT, PET, and MRI, have not been extensively used for biomarker detection. Moreover, a comprehensive evaluation of these imaging techniques, including *in vivo* stability, biosafety, and diagnostic accuracy, is required for their clinical application. Nevertheless, the development of imaging probes for multiple biomarkers is a promising research direction for their clinical application in precise prediction, real-time monitoring, and accurate assessment of therapeutic outcomes of NDDS-aided ICI therapy.

### 5.4. Exploring new models

*In vitro* and *in vivo* models are indispensable tools for evaluating the antitumor efficacy, elucidating the mechanisms, and assessing the safety of ICIs. It has been noticed that the results of preclinical studies are often not replicable in clinical trials. This discrepancy can be ascribed to intrinsic differences between preclinical models and human patients. In order to bridge this gap, advanced models are developed to accurately replicate pathological conditions and tumor progression processes observed in human patients. For i*n vitro* studies, 3D *in vitro* systems have been developed to model immune responses to solid tumors. Thus, they have been used to screen drugs, probe mechanisms, and examine interactions between immune cells and tumor cells since the conventional two-dimensional cell culture method fails to mimic interactions between cells and the ECM, between different types of cells, and/or between the same type of cells 423. These interactions are crucial for regulating immune cell polarization, maintaining phenotypic stability, and influencing tumor cell proliferation and metabolic activity. Immune-oncology models developed with the assistance of biomaterials, such as scaffolds, organoids, and microfluidics, are more sophisticated *in vitro* platforms that can mimic the structural complexity of tumors and preserve tumor heterogeneity 424, 425. These *in vitro* models have been exploited to replicate immune interactions in irAEs-related organs, which can help in the understanding of irAEs and assessing them induced from NDDS-based immunotherapeutic formulations.

Preclinical animal models have also been constructed to bridge the gaps between animal models and human patients. It has been observed that ICIs that are efficacious in animal models may fail in humans, and the incidence of irAEs in animal models is often lower than that in patients 426. To address the challenge of human immune system homeostasis and the interactions between immune cells and tumor cells, a myriad of *in vivo* animal models have been developed to simulate tumor growth, examine immune responses, evaluate drug efficacy, and assess irAEs. Among them, xenograft mice models resemble clinical patients more closely than traditional syngeneic tumor models. The patient-derived xenograft (PDX) model developed by transplanting human tumor tissues into immunodeficient mice preserves the heterogeneity and complexity of the original tumor. The PDX model can be employed for personalized treatment evaluation 427. Humanized mice models are in the development stage to accurately mimic the intricate interaction between the immune system and the tumor 428. Engineered transgenic mice are often constructed with specific gene phenotypes. Autoimmune-prone mice, such as non-obese diabetic (NOD) mice and the Murphy Roths Large (MRL) mice with the lymphoproliferation spontaneous mutation (*Fas*^lpr^), have been utilized as model animals to assess irAEs of immunotherapy. Depletion of immunosuppressive cells, represented by Tregs, in C57BL/6 and BALB/c mice, has been harnessed to predict the occurrence of irAEs under the administration of ICIs 429, 430. Other experimental animals, such as dogs and cynomolgus monkeys, are also important research tools for accelerating the translation of preclinical outcomes of NDDS-based Immunotherapeutics into their clinical benefits. However, these animal models can only partially replicate the dynamic, complex system in patients. Efforts have been driven to reduce the cost and expand their versatility as regular evaluation methods. Moreover, the collection and analysis of clinical samples and processing of large-scale clinical data may provide a better understanding of human pathology, which helps guide the direction of preclinical studies 431. The refinement of current preclinical models and the development of novel models will facilitate successful clinical translation of NDDS-based ICI Immunotherapeutics.

## 6. Conclusion

This review briefly elaborated on the predominant mechanisms for irAEs and ICI resistance. Strategies for designing and formulating nanoplatforms for ICB therapy to improve immunotherapeutic effects and address irAEs and ICI resistance were reviewed. Different types of ICIs that have been encapsulated in NDDSs were summarized, and nanomedicines derived from NDDSs have been demonstrated to enhance the immunogenicity of tumors, strengthen the function of APCs, manipulate suppressive immune cells in the TME, modulate the metabolism of tumor cells, and remodel non-immune components in the TME. Although advanced NDDS-based ICI treatment strategies have shown their effectiveness in preclinical models, very few of them have reached clinical translation. Based on a comprehensive summary of current progress, we analyzed unmet clinical needs and existing challenges in several key areas, including irAEs and drug resistance mechanisms, safety issues stemming from nanomedicine construction, NDDS-based integrated diagnosis and treatment, and *in vitro/in vivo* new models. Our insights into the future direction of research and development of NDDS-based ICB therapy could help accelerate its clinical application and benefit more cancer patients.

## Figures and Tables

**Table 1:** Nanovaccines for combination therapy with ICIs to activate APCs.

Nanovaccine	Antigen	Adjuvant	Carrier	ICI	Cancer model	Refs
OVA@MM	OVA	Mn^2+^	Mn^2+^+2-methylimidazole	aPD-1	B16-OVA melanoma	226
Man-PLL-RT/OVA/CpG	OVA	CpG	Man-PLL-RT (polymer)	shPD-L1	B16-OVA melanoma	227
DGBA-OVA-CpG	OVA	CpG	dendrimer grafted with guanidinobenzoic acid	aPD-1	B16-OVA melanoma	228
PCO	OVA	CpG	protamine	aPD-1	B16-OVA melanoma	207
MALO@HBNS	OVA	STAT3 siRNA	polyester, lipid	aPD-L1	B16-OVA melanoma	199
DC-sEVs-CpG	OVA	CpG	DC-derived small EVs	aPD-1	B16-OVA melanoma	197
DoriVac	OVA	CpG	square-block DNA origami	aPD-L1	B16-OVA melanoma	196
nChap@OVA	OVA	R848	mannose-PEG-*b*-PCL, PAE-*b*-PCL	aPD-1	B16-OVA melanoma	229
PP-SS-OVA/CpG	OVA	CpG	PCL-PEG-PDS, PCL-PEI	aPD-1	B16-OVA melanoma	195
NL(pro-TLR7/8a)	OVA	liposomal prodrug-like TLR7/8 agonist (TLR7/8a)	liposome	aCTLA-4+aPD-1	B16-OVA melanoma	201
P*_D_*-K-OVA	OVA	poly(*L*-phenylalanine)*-block-*poly(*D*-lysine)	poly(*L*-phenylalanine)*-block-*poly(*D*-lysine)	aPD-1	B16-OVA melanoma	198
KK2DP7/OVA	OVA	dendrimer polypeptide	dendrimer polypeptide	aPD-1	E.G7-OVA lymphoma	224
SMONV	OVA	/	soft mesoporous organosilica	aPD-L1	E.G7-OVA lymphoma	213
4RDP(F5)-OVA	OVA	fluorinated supramolecular peptide adjuvant	fluorinated supramolecular peptide adjuvant	aPD-L1	E.G7-OVA lymphoma	223
PoIC/OVA-R8L	OVA	poly I:C	liposome	aPD-L1	MO5 melanoma	194
OVA_PEP_-SLNP@CpG	SIINFEKL	CpG	lipid nanoparticle	aPD-1	E.G7-OVA lymphoma	202
RNA-OG-pOVA	SIINFEKL	RNA origami	RNA origami	aPD-1	B16-OVA melanoma	225
C-25/OVA_257-280_	peptide antigen OVA_257-280_	PEGMA-*co*-BMA-*co*-C7AMA	PEGMA-*co*-BMA-*co*-C7AMA	aPD-L1	B16-OVA melanoma	222
R848@M2pep-MPs_AFP_	alpha-fetoprotein (AFP), OVA	R848	engineered microparticles derived from AFP/OVA-overexpressing macrophages	aPD-1	B16-OVA melanoma, Hepa1-6 hepatocellular carcinoma	218
NV, PNV	OVA, supernatant of tumor abrasive fluid	CpG+Mn^2+^	Mn^2+^+2-methylimidazole	aPD-L1	B16-OVA, B16F10 melanoma	230
CpG&Ag	SIINFEKL, Adpgk	CpG	/	aPD-1	B16-OVA melanoma, MC-38 colorectal cancer	221
sHDL-Ag+PolyICLC	Adpgk neoantigen	polyICLC	synthetic high-density protein nanodiscs	aPD-1	MC-38 colorectal cancer	208
banNV	Adpgk neoantigen	R848+CpG	PEG-PLA, PPT-*g*-PEG	aPD-1	MC-38 colorectal cancer	231
Nanovaccine	Adpgk neoantigen, MUT30	CpG+poly(I:C)	PLGA nanoparticles	aPD-1	MC-38, CT26 colorectal cancer, B16F10 melanoma	203
RGO(CpG)-PEG-(M27+M30)	M27 + M30 peptides	CpG	PEGylated reduced graphene oxide nanosheet	aPD-1	B16F10 melanoma	232
Gel(Vaccine NPs)	M27 + M30 peptides	Bacille Calmette-Guérin (BCG) bacterial cell wall skeleton	PLGA	aPD-L1	B16F10 melanoma	211
Nanovaccine	Tyrp1+M20+M27 peptides	Montanide^TM^ ISA 51	DSPE-PEG_2000_	aPD-1	B16F10 melanoma	209
NTV2	M27+M30+M47+M48	/	polymer-peptide	aPD-L1	B16F10 melanoma	210
Mutation-M33-M47 BDVs	M33+M47	bacteria derived vesicles	bacteria derived vesicles, GM-CSF	aPD-1	B16F10 melanoma	220
8FNs@Trp2	TRP2_181-188_	antimicrobial peptide	*L*-phenylalanine-based poly(ester amide) polymers	aPD-1	B16F10 melanoma	200
TCL@CaCO_3_	tumor cell lysates	dsDNA	CaCO_3_	aPD-1	B16F10 melanoma	233
MSNs@cGAMP@CM-SN21	B16F10 cell membranes	cGAMP	mesoporous silica nanoparticles (MSNs) coated with cell membranes	aPD-1	B16F10 melanoma	214
MSN-CpG@CM	B16F10 cell membranes	CpG	MSNs coated with cell membranes	aCTLA-4	B16F10 melanoma	215
DBE@CCNPs	CD47KO/CRT dual-bioengineered B16F10 cell membranes	CpG	PEI + bioengineered B16F10 cell membranes	aPD-L1	B16F10 melanoma	217
LMP	B16F10 cells	Mn^2+^ + LPS	Mn^2+^ + TA	aPD-L1	B16F10 melanoma	234
Vaccine A + vaccine B	water-insoluble and water-soluble components of tumor tissues/cells	poly(I:C)	PLGA	aPD-1	B16F10 melanoma, 4T1 breast cancer	205
MPE-C	MUCI-derived peptide	CpG	mannosylated Pickering emulsion	aPD-1	B16-MUCI melanoma	235
Neo NV	Zfp142 peptide	/	norovirus S protein nanoparticles	aPD-1	4T1 breast cancer	236
PD1/CD40L-NVs	PD-1/CD40L-overexpressed 4T1 cytomembranes	CD40L	PD-1/CD40L-overexpressed 4T1 cytomembranes	PD-1 on nanovaccines	4T1 breast cancer	237
K-nanoadjuvant	E7-long peptide	timely activating TLR7/8a+poly (I:C)	liposome	aPD-L1	TC-1 lung cancer	238
LDHs-cGAMP	TAAs	cGAMP	layered double hydroxides	aPD-L1	Hepa1-6 hepatocellular carcinoma	204
DIA-NPs	chemotherapy-induced antigens	/	/	aPD-1	CT26 colorectal cancer	206
MON@LA-PDE5i@M	LLC cell membranes	phosphodiesterase-5 inhibitor + NO	MSN coated with LLC cell membranes	aPD-L1	LLC lung cancer	216
NA1C	EBNA1ΔGA_92-327_	CpG	tannic acid (TA)	aPD-L1	EBNA1-TC1 lung cancer	239
cBEV	bacteria-derived EVs	Mn^2+^	bacteria-derived EVs	aPD-L1	MCF-7 breast cancer	219

**Table 2:** List of novel nanoplatform-based strategies for immune checkpoint blockade.

Nanoplatform	Therapeutic agents	NDDS types	Mechanism of ICB	Cancer model	Refs
NPRO	SHP2-targeting PROTAC, protoporphyrin IX	peptide nanoparticle	targeted degradation of SHP2	4T1 breast cancer	372
DdLD NPs	PROTAC of BRD4, DOX	self-assembled nanoparticle	targeted degradation of BRD4	CT26 colorectal cancer	373
CDTACs	PROTAC of PD-L1	carbon-dot	targeted degradation of PD-L1	CT26 colorectal cancer, B16-F10 melanoma	374
2-BP/CPT-PLNs	2-bromopalmitate, CPT	polymer-lipid hybrid nanoparticle	inhibiting PD-L1 palmitoylation inhibiting PD-L1 palmitoylation	B16F10 melanoma	379
FRS/DOX	PD-L1 competitive inhibitor peptide, DOX	peptide nanoparticle	inhibiting PD-L1 palmitoylation inhibiting PD-L1 palmitoylation	CT26 colorectal cancer	380
MHI-TMX@ALB	tamoxifen, MHI	albumin nanoparticle	activating AMPK by inhibiting OXPHOS	4T1 breast cancer, MB49 bladder cancer	383
IR-LND@Lip	lonidamine, radiotherapy	liposome	activating AMPK by inhibiting OXPHOS	4T1 breast cancer	384
IR-TAM@Alb	tamoxifen, radiotherapy	albumin nanoparticle	activating AMPK by inhibiting OXPHOS	4T1 breast cancer, MB49 bladder cancer	385
IR-LND@Alb	lonidamine, IR-68	albumin nanoparticle	activating AMPK by inhibiting OXPHOS	CT26 colorectal cancer, MB49 bladder cancer	386
TPP-LND@Lip	lonidamine, radiotherapy	liposome	activating AMPK by inhibiting OXPHOS	LLC lung cancer	387
V(Hb)@DOX	hemoglobin, DOX	biomimetic nanovesicle	alleviating hypoxia	4T1 breast cancer, CT26 colorectal cancer	388
p-Adv-CAT-KR	catalase, KillerRed, adenovirus	adenovirus system	alleviating hypoxia	AKT/YapS127A cholangiocarcinoma	389
mPDAase	hyaluronidase, mesoporous polydopamine	polydopamine nanoparticle	alleviating hypoxia	4T1 breast cancer	390
γ-Fe_2_O_3_/ISDN@(NBCF-CMCS)	γ-Fe_2_O_3_, isosorbide dinitrate	polysaccharide micelle	alleviating hypoxia	H22 hepatoma	391
BMIP_2_N NPs	MnO_2_, IR780, NLG919, PTX	manganese dioxide albumin nanoparticle	alleviating hypoxia	4T1 breast cancer	392
BMI	MnO_2_, IPI549	albumin nanoparticle	alleviating hypoxia	4T1 breast cancer	393
Melanin/MnO_x_ nanohybrids	melanin, MnO_x_	hybride nanoparticle	alleviating hypoxia	4T1 breast cancer	394
Pt@PEG-Ce6	mesoporous Pt, Ce6	Pt nanoparticle	alleviating hypoxia	4T1 breast cancer	395
LipoCu-OA/ACF	acriflavine, copper oleate	liposome	HIF-1 inhibition	4T1 breast cancer	396
PNEs	BAY87-2243, neutrophils	polysaccharide nanoparticle	HIF-1 inhibition	4T1 breast cancer	397
G5C3	ceramide, paclitaxel/carboplatin	liposome	HIF-1 inhibition	LLC-1, H1299 lung cancer	398
QFN	quercetin, Ferrum ion	phenolic-metal nanoparticle	quercetin reduced PD-L1	B16F10 melanoma	399
MB@Bu@MnO_2_	methylene blue (MB), Butformin (Bu)	manganese dioxide albumin nanoparticle	MB inhibited PD-1 signaling and Bu enhanced AMPK phosphorylation	MB49 bladder cancer, 4T1 breast cancer	400
sAZD1080	AZD1080	silicasome	downregulating PD-1 expression by inhibiting glycogen synthase kinase 3	MC38, CT26 colorectal cancer, LLC lung cancer, KPC pancreatic cancer	401
LMDFP	Cu^2+^, D-penicillamine, lanthanide-doped nanocrystals	MSN	Cu^2+^ inhibited PD-L1 expression	PC-3 prostatic cancer, 4T1 breast cancer	402
OA@CuMnCs	Cu^2+^, Mn^2+^, oncolytic adenovirus	biomineralized adenovirus	Cu^2+^ inhibited PD-L1 expression	CT26 colorectal cancer	403
RCH NPs	roscovitine, hemin, celecoxib	albumin nanoparticle	roscovitine suppressed IFN-γ-dependent PD-L1 gene transcription by inhibiting the Cdk5 pathway	B16F10 melanoma	404
CMArg@Lip	ML385, L-arginine, Ce6	liposome	ML385 inhibited the nuclear factor erythroid 2-related factor 2 (NRF2) and NO reduced PD-L1 expression	QBC-939 cholangiocarcinoma	405
MPB-NO@DOX+ATRA	NO, DOX, Mn^2+^, all-trans retinoic acid	prussian blue nanoparticle	NO restrained PD-L1 expression	4T1 breast cancer	406
mCGYL-LOx	lactate oxidase, CuO, Gd_2_O_3_	metal nanoparticle	lactate exhaustion reduced PD-L1 expression	Renca renal cell carcinoma	407
Cu-DBCO/CL	Cu-DBCO nanozyme, cholesterol, lysyl oxidase inhibitor	MOF	cholesterol depletion downregulated the expression of PD-1 and TIM-3	4T1 breast cancer	408
CCP@DA	the cell-membrane targeting chimeric peptide C_16_-Cypate-RRKK-PEG_8_-COOH, diclofenac	peptide nanoparticle	downregulating the PD-L1 expression by destroying the cell membrane and blocking the COX-2/PGE_2_ pathway	4T1 breast cancer	409
a-LDH@YW3-56-PEG	YW3-56, CoW-layered double hydroxide	nanosheet	inhibiting peptidyl arginine deiminase 4	4T1 breast cancer	410

## Abbreviations

ICIs: Immune checkpoint inhibitors
irAEs: immune-related adverse events
ICB: immune checkpoint blockade
NDDSs: Nano drug delivery systems
CTLA-4: cytotoxic T lymphocyte-associated protein 4
PD-1: programmed cell death protein 1
PD-L1: programmed death-ligand 1
ICPs: immune checkpoints
LAG-3: lymphocyte activation gene-3
TIM-3: T cell immunoglobulin and mucin-domain containing-3
TIGIT: T cell immunoglobin and ITIM domain
VISTA: V-domain Ig suppressor of T cell activation
APCs: antigen presenting cells
CTLs: cytotoxic T lymphocytes
GLUT1: glucose transporter 1
BBB: blood-brain barrier
Tregs: regulatory T cells
GBM: glioblastoma
NPs: nanoparticles
ROS: reactive oxygen species
TAMs: tumor-associated macrophages
MDSCs: myeloid-derived suppressor cells
CAFs: cancer-associated fibroblasts
ECM: extracellular matrix
VEGF: vascular endothelial growth factor
TGF-β: transforming growth factor-β
ICD: immunogenic cell death
DAMPs: damage-associated molecular patterns
HMGB1: high mobility group protein B1
CRT: calreticulin
ATP: adenosine triphosphate
PTT: photothermal therapy
PDT: photodynamic therapy
SDT: sonodynamic therapy
CDT: chemodynamic therapy
GSH: glutathione
PTX: paclitaxel
IFN-γ: interferon gamma
DOX: doxorubicin
TAAs: tumor-associated antigens
LND: lonidamine
GSDM: gasdermin
TCA: tricarboxylic acid
OXPHOS: oxidative phosphorylation
ER: endoplasmic reticulum
MHC: major histocompatibility complex
DNMT: DNA methyltransferase
HDAC: histone deacetylase
HMT: histone methyltransferase
TLR: toll-like receptor
cGAS: cyclic guanosine monophosphate-adenosine monophosphate synthase
STING: stimulator of interferon genes
CDNs: cyclic dinucleotides
TNBC: triple-negative breast cancer
dsDNA: double-stranded DNA
mtDNA: mitochondrial DNA
OVA: Ovalbumin
Teffs: effector T cells
HCC: hepatocellular carcinoma
LDHA: lactate dehydrogenase A
IDO: indoleamine 2,3-dioxygenase
AA: arachidonic acid
PGE_2_: prostaglandin E_2_
COX-2: cyclooxygenase-2
NSCLC: non-small cell lung cancer
IFP: interstitial fluid pressure
HAase: hyaluronidase
PROTAC: proteolysis targeting chimera
AMPK: adenosine monophosphate-activated protein kinase
MSI: microsatellite instability
TMB: tumor mutation burden
dMMR: mismatch repair deficiency
